# DeepST: identifying spatial domains in spatial transcriptomics by deep learning

**DOI:** 10.1093/nar/gkac901

**Published:** 2022-10-17

**Authors:** Chang Xu, Xiyun Jin, Songren Wei, Pingping Wang, Meng Luo, Zhaochun Xu, Wenyi Yang, Yideng Cai, Lixing Xiao, Xiaoyu Lin, Hongxin Liu, Rui Cheng, Fenglan Pang, Rui Chen, Xi Su, Ying Hu, Guohua Wang, Qinghua Jiang

**Affiliations:** School of Life Science and Technology, Harbin Institute of Technology, Harbin 150000, China; School of Life Science and Technology, Harbin Institute of Technology, Harbin 150000, China; Department of Neuropharmacology and Novel Drug Discovery, School of Pharmaceutical Sciences, Southern Medical University, Guangzhou 510515, China; Center for Brain Science and Brain-Inspired Intelligence, Guangdong-Hong Kong-Macao Greater Bay Area, Guangdong 523335, China; School of Life Science and Technology, Harbin Institute of Technology, Harbin 150000, China; School of Life Science and Technology, Harbin Institute of Technology, Harbin 150000, China; School of Life Science and Technology, Harbin Institute of Technology, Harbin 150000, China; School of Life Science and Technology, Harbin Institute of Technology, Harbin 150000, China; School of Life Science and Technology, Harbin Institute of Technology, Harbin 150000, China; School of Life Science and Technology, Harbin Institute of Technology, Harbin 150000, China; School of Life Science and Technology, Harbin Institute of Technology, Harbin 150000, China; School of Life Science and Technology, Harbin Institute of Technology, Harbin 150000, China; School of Life Science and Technology, Harbin Institute of Technology, Harbin 150000, China; School of Life Science and Technology, Harbin Institute of Technology, Harbin 150000, China; Department of Forensic Medicine, Guangdong Medical University, Dongguan 523808, China; ChinaFoshan Maternity & Child Healthcare Hospital, Southern Medical University, Foshan 528000, China; School of Life Science and Technology, Harbin Institute of Technology, Harbin 150000, China; School of Computer Science and Technology, Harbin Institute of Technology, Harbin 150000, China; School of Life Science and Technology, Harbin Institute of Technology, Harbin 150000, China; School of Interdisciplinary Medicine and Engineering, Harbin Medical University, Harbin 150076, China

## Abstract

Recent advances in spatial transcriptomics (ST) have brought unprecedented opportunities to understand tissue organization and function in spatial context. However, it is still challenging to precisely dissect spatial domains with similar gene expression and histology *in situ*. Here, we present DeepST, an accurate and universal deep learning framework to identify spatial domains, which performs better than the existing state-of-the-art methods on benchmarking datasets of the human dorsolateral prefrontal cortex. Further testing on a breast cancer ST dataset, we showed that DeepST can dissect spatial domains in cancer tissue at a finer scale. Moreover, DeepST can achieve not only effective batch integration of ST data generated from multiple batches or different technologies, but also expandable capabilities for processing other spatial omics data. Together, our results demonstrate that DeepST has the exceptional capacity for identifying spatial domains, making it a desirable tool to gain novel insights from ST studies.

## INTRODUCTION

Tissue is composed of diverse cells whose spatial organization is of high importance to exert their biological functions. Recent advancements in spatial transcriptome (ST), such as the 10 × Visium (https://www.10xgenomics.com/), Slideseq (1,2) and Stereoseq (3), make it possible to understand the tissue functions and cellular architectures on transcriptomic level via sequencing *in situ*.

Identifying spatial domain (i.e. a region that are spatially coherent in both gene expression and histology) is one of the most important topics in spatial transcriptomics. At present, the methods to identify spatial domains could be mainly divided into two categories, non-spatial and spatial clustering methods. Traditional non-spatial clustering algorithms, such as K-means and Louvain (4), take gene expression data as input, resulting in clusters that hardly correspond with tissue sections. On the other hand, spatial clustering methods that combine gene expression, spatial location, and morphology have been developed to account for the spatial dependency of gene expression to match spatial location better. BayesSpace (5) adopts a fully Bayesian statistical method using a spatial prior to encourage nearby locations to belong to the same cluster. stLearn (6) offers a within-tissue normalization technique that normalizes gene expression using morphological distance based on characteristics collected from morphology images (e.g. by hematoxylin and eosin (H&E) staining) and spatial locations. SpaGCN (7) combines gene expression, spatial location, and morphology data to identify spatial domains by generating an undirected weighted graph that captures the spatial dependency of the data. SEDR (8) employs a deep auto-encoder network and a graph auto-encoder to embed spatial information. Although these algorithms can identify spots or cells into distinct domains, they mainly depend on linear principal component analysis to extract the highly variable characteristics of gene expression, which involves a linear transformation, so they are unable to model complicated nonlinear interactions. Even existing methods can provide some useful information, these tools often do not take full advantage of spatial information and are limited in predicting tissue architectures. In addition, most spatial methods for analyzing numerous ST data cannot properly correct batch effects, and their inability to process other spatial omics data (9,10) makes them less versatile. Overall, it is still challenging to accurately identify spatial domains from ST data.

Herein, we proposed a customizable deep learning framework for ST (DeepST) to accurately identify spatial domains. DeepST extracts feature vectors from morphological image tiles using a pre-trained deep neural network model, then integrates the extracted features with gene expression and spatial location data to characterize the correlation of spatially adjacent spots, and creates a spatial augment gene expression matrix. DeepST uses a graph neural network (GNN) autoencoder and a denoising autoencoder to jointly generate a latent representation of augmented ST data, while domain adversarial neural networks (DAN) are used to integrate ST data from multiple batches or different technologies. We performed extensive tests and comparisons with existing algorithms on ST data generated by different platforms (e.g. 10 × Visium, SlideseqV2 (2), and Stereoseq (3)) as benchmarks. DeepST can also process imaging-based molecular data (e.g. MERFISH (11), 4i (9) and MIBI-TOF (10)), in particular, three-dimensional (3D) expression domains are extracted on MERFISH data. Further testing on a breast cancer ST dataset, DeepST discerned heterogeneous sub-regions within the visually homogenous tumour region that have not been detected in traditional intratumoral results. Taken together, our results demonstrated that DeepST is of great power in the accurate identification of spatial domains, also scalable in processing additional spatial omics data.

## MATERIALS AND METHODS

### Spatial data augmentation

Transcriptome-wide gene expression profiles with extra spatial location information and tissue morphology are provided by spatial gene expression technologies. DeepST uses these two extra tissue data to augment gene expression across adjacent spots. Specifically, DeepST assesses gene expression similarity, morphological similarity, and spatial neighbours between spots:

1. Correlation ${\bf{G}}{{\bf{C}}_{ij}}$ was applied to calculate the weights of spatial gene expression between spot ${{\bf{S}}_i}$ and spot ${{\bf{S}}_j}$ as:
  (1) $$\begin{equation*}{\bf{G}}{{\bf{C}}_{ij}} = 1 - \frac{{\left( {{{\bf{S}}_i} - \overline {{{\bf{S}}_i}} } \right) \cdot \left( {{{\bf{S}}_j} - \overline {{{\bf{S}}_j}} } \right)}}{{{{\left\| {\left( {{{\bf{S}}_i} - \overline {{{\bf{S}}_i}} } \right)} \right\|}_2}{{\left\| {\left( {{{\bf{S}}_j} - \overline {{{\bf{S}}_j}} } \right)} \right\|}_2}}}.\end{equation*}$$
2. For ST data with morphological information, we first segmented an image (H&E staining tiles) according to the coordinates of each spot to obtain its partial image. Then use *torchvision.transforms* (12) function to transform and augment partial images, including normalizing, rotating, adjusting sharpness, etc. The high-level features of each spot tile are extracted from a pretrained convolutional neural network (optional; default is Inception v3 (13)) model that can transform each spot image into 2048-dimensional latent variables. To better represent the spot morphology, we performed principal component analysis (PCA) to extract the first 50 principal components (PCs) (optional) as latent characteristics. Finally, the weights of morphological similarity ${\bf{M}}{{\bf{S}}_{ij}}$ between spot ${{\bf{S}}_i}$ and adjacent spot ${{\bf{S}}_j}$ were calculated using the cosine distance as:
  (2) $$\begin{equation*}{\bf{M}}{{\bf{S}}_{ij}} = 1 - \frac{{{{\bf{S}}_i} \cdot {{\bf{S}}_j}}}{{{{\left\| {{{\bf{S}}_i}} \right\|}_2}{{\left\| {{{\bf{S}}_j}} \right\|}_2}}}.\end{equation*}$$
3. We used spatial coordinates to determine the distance between each spot and all other spots, then ordered the distances between the top 4 (optional) adjacent spots to count the radius $\gamma$ (mean add variance). For a given spot ${{\bf{S}}_i}$, a spot ${{\bf{S}}_j}$ is considered to be a neighbour, then ${\bf{S}}{{\bf{W}}_{ij}} = 0$ if and only if the distance between two spots is less than $\gamma$, otherwise ${\bf{S}}{{\bf{W}}_{ij}} = 1$.

DeepST then enhances gene expression $\mathop {{\bf{G}}{{\bf{E}}_i}}\limits^ \sim$ of each spot ${{\bf{S}}_i}$ incorporating gene expression correlation, spatial neighbour, and morphological similarity as:

(3) $$\begin{equation*}\mathop {{\bf{G}}{{\bf{E}}_i}}\limits^ \sim = {\bf{G}}{{\bf{E}}_i} + \frac{{\sum\limits_{j = 1}^n {{\bf{G}}{{\bf{E}}_j} \cdot {\bf{M}}{{\bf{S}}_{ij}} \cdot {\bf{G}}{{\bf{C}}_{ij}} \cdot {\bf{S}}{{\bf{W}}_{ij}}} }}{n},\end{equation*}$$

if in 10 × Visium, otherwise as:

(4) $$\begin{equation*}\mathop {{\bf{G}}{{\bf{E}}_i}}\limits^ \sim = {\bf{G}}{{\bf{E}}_i} + \frac{{\sum\limits_{j = 1}^n {{\bf{G}}{{\bf{E}}_j} \cdot {\bf{G}}{{\bf{C}}_{ij}} \cdot {\bf{S}}{{\bf{W}}_{ij}}} }}{n},\end{equation*}$$

where ${\bf{G}}{{\bf{E}}_i}$ and ${\bf{G}}{{\bf{E}}_j}$ are the raw gene expressions for spot ${{\bf{S}}_i}$ and *n* adjacent spots ${{\bf{S}}_j}$.

### Graph construction

To combine the similarity of the adjacent spots of a given spot, DeepST uses spatial coordinates to calculate the distances between spots (optional; default is BallTree (14)), and constructs a cell–cell spatial relationship graph using the top 12 (optional) nearest neighbours. If ${\bf{A}}$ is the adjacency matrix, then the value ${{\bf{A}}_{ij}} = {{\bf{A}}_{ji}} = 1$ at spot $i$ and spot $j$ means that $i$ and $j$ are neighbors, otherwise ${{\bf{A}}_{ij}} = 0$. Self-loops are added into each spot.

### Denoising autoencoder

DeepST implements a denoising autoencoder for the latent representation of gene expression using linear layers with PyTorch (12). The encoder $E$, which consists of multiple fully connected stacked linear layers as set by a user, converts the integrated gene expressions ${\bf{X}}$ (the PCA embeddings of $\mathop {{\bf{G}}{{\bf{E}}_i}}\limits^ \sim$) into a low-dimensional representation ${{\bf{Z}}_g}$ as:

(5) $$\begin{equation*}E({\bf{X}}) = {{\bf{Z}}_g},{\bf{X}} \in {R^{N \times M}},{{\bf{Z}}_g} \in {R^{N \times R}},\end{equation*}$$

where $N$ is the number of spots, $M$ is the number of input genes, and $R$ is the dimension of the last encoder layer. Conversely, the decoder $D$ reverses the latent representation and tries to reconstruct the original input as:

(6) $$\begin{equation*}\begin{array}{@{}l@{}} {{\bf{Z}}_{g^{\prime}}} = {{\bf{Z}}_g} + {\bf{Z}},\\ D({{\bf{Z}}_{g^{\prime}}}) = {\bf{X^{\prime}}},{{\bf{Z}}_{g^{\prime}}} \in {{\rm{R}}^{N \times (R + R^{\prime})}},{\bf{X^{\prime}}} \in {R^{N \times M}}, \end{array}\end{equation*}$$

where ${\bf{X^{\prime}}}$ is the reconstructed gene expression matrix, and ${\rm{N}}$, ${\rm{M}}$ and ${\rm{R}}$are the same as above, ${{\bf{G}}_g}$ is the spatial embedding learned by GNN encoder and $R^{\prime}$ is the final layer dimension. The mean squared error is applied to determine how comparable the input gene and reconstructed expressions are as:

(7) $$\begin{equation*}{L_l} = \frac{1}{N}\sum\limits_{i = 1}^N {{{\left\| {{{\bf{X}}_i} - D\left( {E\left( {{{\bf{X}}_i}} \right)} \right)} \right\|}^2}} .\end{equation*}$$

### Variational graph autoencoder

The encoder (inference model) of the variational graph autoencoder is composed of GNNs (optional) based on PyG (15), where a user can choose GCNConv (default), graph attention network, among others. DeepST takes an adjacency matrix ${\bf{A}}$ and a feature matrix ${\bf{X}}$ (the PCA embeddings of $\mathop {{\bf{G}}{{\bf{E}}_i}}\limits^ \sim$) as inputs, and generates the graph embedding ${\bf{Z}}$ as output. The first two GNN (optional) layers generate a lower-dimensional feature matrix, which is defined as:

(8) $$\begin{equation*}\begin{array}{@{}l@{}} {\bf{\bar X}} = GNN({\bf{X}},{\bf{A}}) = {\bf{\tilde A}}{\rm{ReLU}}({\bf{\tilde AX}}{{\bf{W}}_{\bf{0}}}){{\bf{W}}_1},\\ {\bf{\tilde A}} = {{\bf{D}}^{ - \frac{1}{2}}}{\bf{{\rm A}}}{{\bf{D}}^{ - \frac{1}{2}}}, \end{array}\end{equation*}$$

where ${\bf{\tilde A}}$ is the symmetrically normalized adjacency matrix. The last GNN layer generates $\mu$ and $\log {\sigma ^2}$, where

(9) $$\begin{equation*}\begin{array}{@{}l@{}} \mu = GN{N_u}({\bf{X}},{\bf{A}}) = {\bf{\tilde A\bar X}}{{\bf{W}}_{\bf{2}}},\\ \log {\sigma ^2} = GN{N_\sigma }({\bf{X,A}}) = {\bf{\tilde A\bar X}}{{\bf{W}}_{\bf{2}}}, \end{array}\end{equation*}$$

Specifically, $GN{N_u}$ and $GN{N_\sigma }$ share ${{\bf{W}}_1}$, but ${{\bf{W}}_{\bf{2}}}$ is different. Then, Z can be calculated using a parameterization trick as:

(10) $$\begin{equation*}{\bf{Z}} = \mu + \log {\sigma ^2} * \varepsilon ,\end{equation*}$$

where $\varepsilon \sim N( {0,1} )$. The decoder (generative model) is defined by a simple inner product between latent variable ${\bf{Z}}$. The reconstructed adjacency matrix is generated by calculating the probability of an edge between two spots in pairs as:

(11) $$\begin{equation*}\begin{array}{@{}l@{}} p({\bf{A|Z}}) = \prod\limits_{i = 1}^N {\prod\limits_{j = 1}^N {p({{\bf{A}}_{ij}}|{z_i},{z_j})} } ,\\ p({{\bf{A}}_{ij}} = 1|{z_i},{z_j}) = \sigma ({\bf{Z}}{{\bf{Z}}^{\bf{T}}}), \end{array}\end{equation*}$$

where $\sigma ( \cdot )$ is a logistic sigmoid function.

The loss function includes the reconstruction loss between the generated graph and the original graph, and the Kullback–Leibler divergence of the node representation vector distribution and the normal distribution as:

(12) $$\begin{equation*}{L_g} = {E_{q({\bf{Z|X,A}})}}[\log p({\bf{A|Z}})] - KL[q({\bf{Z|X,A}})\left\| {p({\bf{Z}})} \right.],\end{equation*}$$

where ${E_{q({\bf{Z|X,A}})}}[\log p({\bf{A|Z}})]$ is the binary cross-entropy function, $p( {\rm{Z}} ) = \prod\limits_i {N(0,I)}$.

### Domain adversarial neural networks

The purpose of DAN (16) is to map the source and target domains of different distributions into the same feature space, so that the distance in the space is as close as possible. DAN includes feature extractor, and domain classifier. Among them, the feature extractor is composed of a joint linear layer and a graph neural network. We add a domain discriminator, which is connected by a gradient reversal layer (GRL) in the middle. A domain classification layer ${G_d}$ learns a function ${G_f}:Z_g^{\prime} \to {R^D}$ that maps an example into a new $D$dimensional representation, and is parameterized by a matrix-vector pair $(W,b) \in {R^{D \times m}} \times {R^D}$:

(13) $$\begin{equation*}{G_d}(x;W,b) = sigm(Wx + b),\end{equation*}$$

with $sigma(a) = [ {\frac{1}{{1 + \exp ( - {a_i})}}} ]_{{\rm{ }}i = 1}^{{\rm{ }}| a |}$. We define its loss by

(14) $$\begin{equation*}{L_d} = - \frac{1}{N}\sum\limits_i {\sum\limits_{d = 1}^M {{D_{id}}\log ({p_{id}})} } ,\end{equation*}$$

where $M$ is the number of domains, ${D_{id}}$ is the sign function, if the true label of sample $i$ is equal to $d$, take 1, otherwise take 0, and${p_{id}}$ is the probability that the observed sample $i$ belongs to category $d$.

### Spatial data integration

Different from the framework of spatial domain recognition, DeepST integrates spatial data through a domain adversarial framework. We have given multi-batches or spatial platform datasets domain labels, and then train the model through GRL. We compare the spatial methods SEDR and stLearn on the DLPFCs dataset. Integrating different spatial platform datasets, we compared DeepST with a variety of methods that have been widely used in single-cell data integration, including Harmony (17) and Scanorama (18). The specific parameter settings and codes of these methods can be found in the Supplementary Notes. All other parameters were kept at default values. We did not utilize the correct function, as this included both preprocessing and integration of the data. For more equitable comparisons, we tried to use the same preprocessing pipelines for all methods and only compared only the integration steps.

### Data preprocessing and dimension reduction

We began by removing areas outside the primary tissue region from all datasets. Using the Scanpy package (19), raw gene expression data were filtered, log-transformed, and standardized according to library size. DeepST uses PCA for dimension reduction on augmented gene expression data, and the dimensionality reduction data are used as input for the next model training.

### Benchmarking

After data preprocessing, Seurat runs PCA to extract the top 30 PCs and locate adjacent spots. The clusters are then identified using the Louvain clustering technique. Other approaches use the same number of domains as the truth layers and use the suggested settings (only for DLPFCs). In the original paper, the authors suggested the parameters used to build spatial clustering algorithms (BayesSpace (5), SpaGCN (7), stLearn (6) and SEDR (8); Supplementary Notes). The adjusted rank index (ARI) (20) is used to compare the performances of different clustering techniques on datasets containing spot-type labels. The characteristics of ARI are:

(15) $$\begin{eqnarray*}ARI\left( {{{\bf{P}}^{\bf{*}}}{\bf{,P}}} \right) &=& \frac{{\sum\limits_{ij} {\left( {\begin{array}{@{}*{1}{c}@{}} {{N_{ij}}}\\ 2 \end{array}} \right) - {{\left[ {\sum\limits_i {\left( {\begin{array}{@{}*{1}{c}@{}} {{N_i}}\\ 2 \end{array}} \right)} + \sum\limits_j {\left( {\begin{array}{@{}*{1}{c}@{}} {{N_j}}\\ 2 \end{array}} \right)} } \right]} \mathord{\left/ {\vphantom {{\left[ {\sum\limits_i {\left( {\begin{array}{@{}*{1}{c}@{}} {{N_i}}\\ 2 \end{array}} \right)} + \sum\limits_j {\left( {\begin{array}{@{}*{1}{c}@{}} {{N_j}}\\ 2 \end{array}} \right)} } \right]} {\left( {\begin{array}{@{}*{1}{c}@{}} N\\ 2 \end{array}} \right)}}} \right. } {\left( {\begin{array}{@{}*{1}{c}@{}} N\\ 2 \end{array}} \right)}}} }}{{\frac{1}{2}\left[ {\sum\limits_i {\left( {\begin{array}{@{}*{1}{c}@{}} {{N_i}}\\ 2 \end{array}} \right)} + \sum\limits_j {\left( {\begin{array}{@{}*{1}{c}@{}} {{{\rm{N}}_j}}\\ 2 \end{array}} \right)} } \right] - {{\left[ {\sum\limits_i {\left( {\begin{array}{@{}*{1}{c}@{}} {{N_i}}\\ 2 \end{array}} \right)} + \sum\limits_j {\left( {\begin{array}{@{}*{1}{c}@{}} {{N_j}}\\ 2 \end{array}} \right)} } \right]} \mathord{\left/ {\vphantom {{\left[ {\sum\limits_i {\left( {\begin{array}{@{}*{1}{c}@{}} {{N_i}}\\ 2 \end{array}} \right)} + \sum\limits_j {\left( {\begin{array}{@{}*{1}{c}@{}} {{N_j}}\\ 2 \end{array}} \right)} } \right]} {\left( {\begin{array}{@{}*{1}{c}@{}} N\\ 2 \end{array}} \right)}}} \right. } {\left( {\begin{array}{@{}*{1}{c}@{}} N\\ 2 \end{array}} \right)}}}},\nonumber\\ \end{eqnarray*}$$

where $N$ is the number of spots, ${N_{ij}}$ is the number of spots of class label $C_j^ * \in {P^ * }$ assigned to cluster ${C_i}$ in partition $P$, and ${N_i}/{N_j}$ is the number of spots in cluster ${C_i}/{C_j}$ of partition$P$. A high ARI ($ARI \in [0,1]$) score indicates a good performance.

### Clustering metrics

If spatial domain annotations are not available, we compare two commonly used clustering metrics, the Silhouette Coefficient (SC) score and Davies-Bouldin (DB) score. SC is calculated using the mean intra-cluster distance $a$ and the mean nearest-cluster distance $b$ for each sample. The Silhouette Coefficient score for a sample is ${{(b - a)} \mathord{/ {\vphantom {{(b - a)} {\max (a,b)}}} } {\max (a,b)}}$, and the best value is 1 and the worst value is –1. DB is defined as the average similarity measure of each cluster with its most similar cluster, where similarity is the ratio of within-cluster distances to between cluster distances. The minimum score is zero, with lower values indicating better clustering. We use the tool sklearn (14) to calculate these two metrics. In this paper, BayesSpace and SpaGCN have no latent variable output and cannot calculate DB and SC values.

### Clustering and visualization

On the basis of DeepST embeddings, we used the leiden method (in Scanpy (19)) to identify spatial domains. DeepST finds the best resolution in two ways. (i) When the number of spatial domains is known *a priori*, resolutions are achieved by grid searching between 0.1 and 2.5, with a step size of 0.01, until the necessary number of clusters is reached. (ii) When there is no prior knowledge, DeepST uses grid search to traverse resolutions between 0.1 and 2.5, with a step size of 0.01, and at the same time calculate Calinski and Harabasz (CH) score (known as variance ratio criterion) using sklearn (14), finally determine the resolution at the highest CH value. For visualization, the uniform manifold approximation and projection (UMAP) was used.

### Identification and functional analysis of differentially expressed genes (DEGs)

For brain datasets and Stereo-seq, we used the Wilcoxon test in Scanpy to find DEGs for each spatial domain with 1% false discovery rate threshold. We used limma (21) to identify DEGs in breast cancer datasets, and genes with |log fold change|$ \ge$2 were used as input for gene ontology enrichment analysis using clusterProfiler (22). Enriched terms with positive or negative z-scores were plotted.

## RESULTS

### Overview of the DeepST workflow

**Figure 1.**
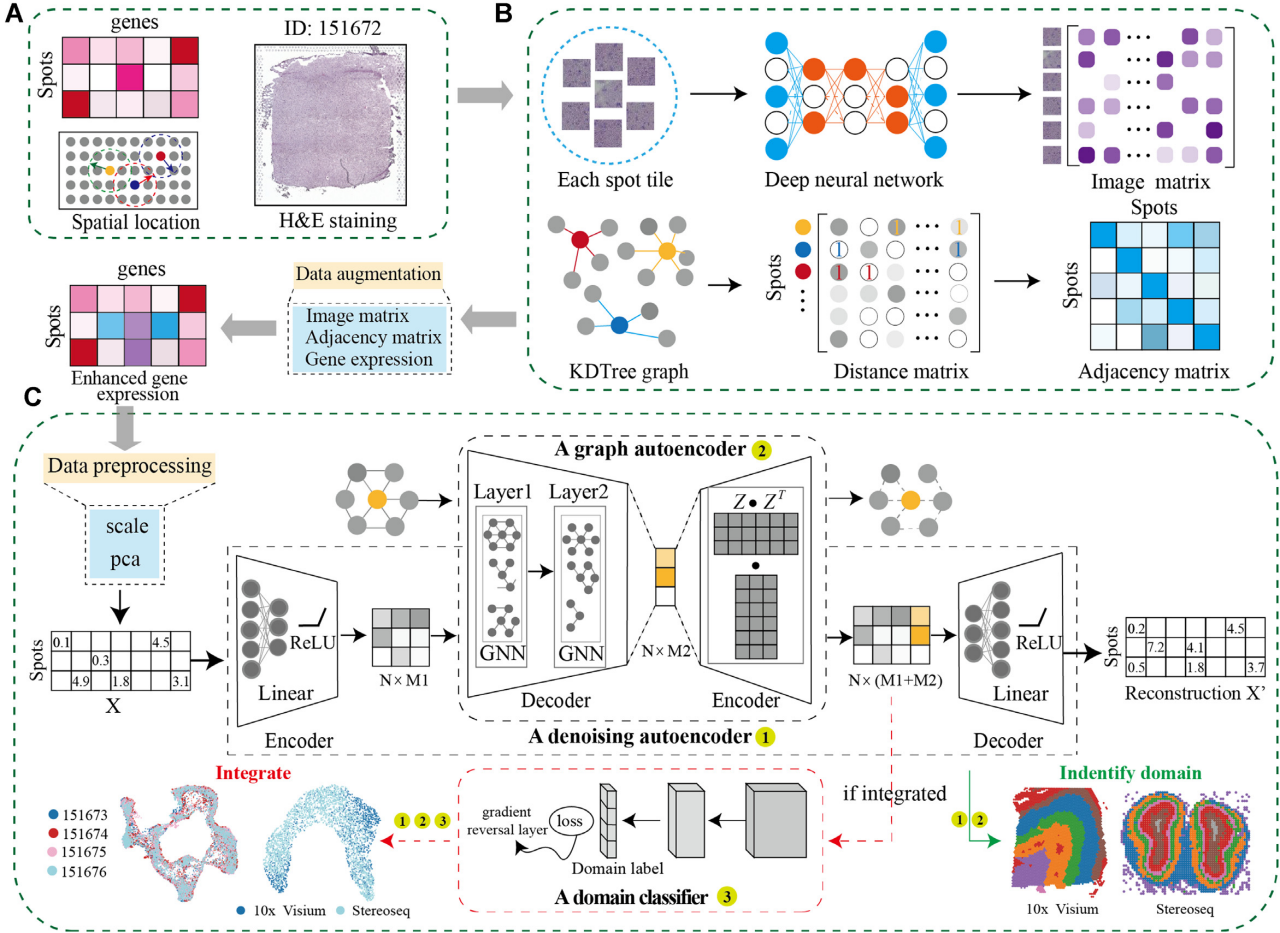
Workflow of the DeepST algorithm. (**A**) DeepST workflow begins with ST data, taking hematoxylin and eosin (H&E) staining (optional), spatial coordinates, and spatial gene expression as input. (**B**) DeepST initially uses the H&E staining to collect tissue morphological information, then normalizes the gene expression of each spot based on similarity against adjacent spots using a pre-trained deep learning model. Morphological similarity between adjacent spots is calculated by this matrix, and the weights of gene expression and spatial location are merged to re-assign an augmented expression value for each gene inside a spot. (**C**) DeepST generates three network frameworks, where a denoising autoencoder network and a variational graph autoencoder are used to extract the final latent embeddings, and a domain discriminator is used to fuse spatial data from various distributions (red dotted box, the part only for integration tasks).

DeepST characterizes spatial domains by modeling a low-dimensional representation of integrating gene expression, spatial location, and tissue morphology information (Figure 1A). To establish a morphological feature matrix, tissue topography data from H&E staining is first processed by a pre-trained deep learning network. Combined with morphological features and spatial neighbor information, the gene expression of each spot is enhanced (Figure 1B). Then, a denoising autoencoder is employed to learn a nonlinear mapping from integrated feature space to a low-dimensional representation space to reduce model overfitting. Simultaneously, DeepST computes a graph adjacency matrix based on spatial coordinates by k-nearest neighbours. A variational graph autoencoder is inserted into the same framework to map spatial associations of spots, thereby generating spatial embedding via integrated representation with the corresponding spatial adjacent spots (Figure 1C; see Materials and Methods for details). The final latent embeddings are formed by concatenating the integrated representation and spatial embedding. If the submission task is to integrate several spatial platforms or multi-batches, latent embeddings will be fed into a domain discriminator connected by a gradient reversal layer (Figure 1C, red dotted box). These latent embeddings can be used to identify spatial domains, correct batch effects and perform downstream analysis.

### Benchmarking of DeepST against state-of-the-art methods

**Figure 2.**
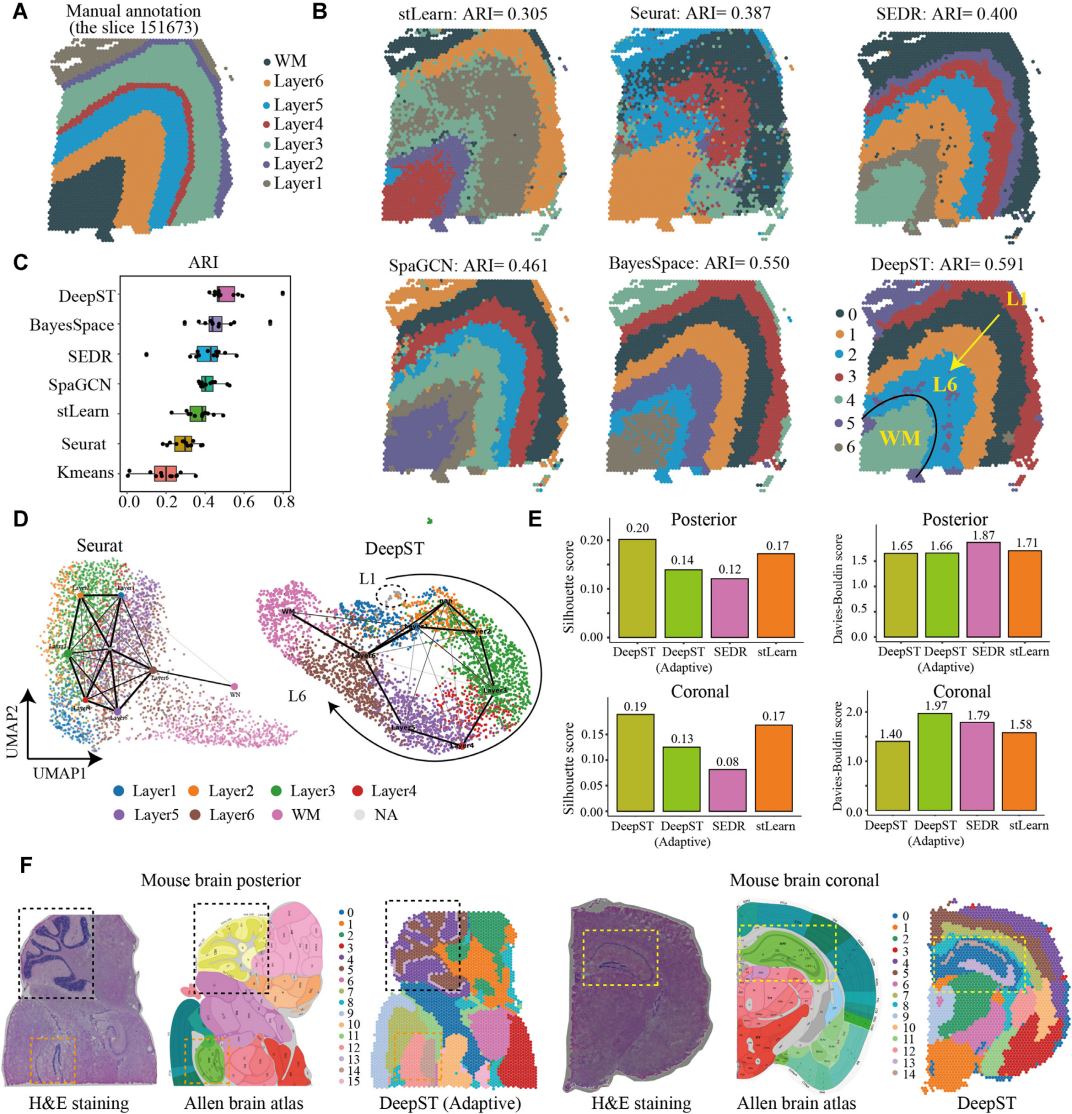
DeepST improves spatial domain recognition in brain tissue. (**A**) DLPFC layers were annotated by Maynard et al.(23). The ground truth of spots was mapped on their spatial position in slide 151673, which was separated into six cortical layers (L1–L6) and white matter (WM). Layers with annotations are provided on the remaining slides (Supplementary Figures 2–12). (**B**) Identification of spatial domains by DeepST, and existing state-of-the-art algorithms (BayesSpace, SpaGCN, SEDR, stLearn, Seurat and K-means) algorithms for slide 151673. (**C**) Boxplot of the performance of DeepST and other algorithms for all 12 DLPFCs. The x-axis shows the adjusted rand index (ARI), which was used to compare the similarity of the predicted spatial layers and the manually annotated layers for each algorithm. (**D**) UMAP visualizations and PAGA graphs were generated for slide 151673 with Seurat-derived principal components (left) and DeepST-derived embeddings (right). (**E**) Histograms of Silhouette Coefficient (SC) and Davies-Bouldin (DB) scores for mouse brain posterior and coronal data, respectively, including algorithms DeepST, DeepST (Adaptive, means no prior knowledge), SEDR and stLearn. (**F**) Spatial domains of mouse brain tissue sagittal posterior and coronal regions. The H&E staining generated from raw data (left); The corresponding anatomical Allen Mouse Brain Atlas (middle, https://atlas.brain-map.org/); Spatial domains identified by DeepST (right). The yellow box denotes the cornu ammonis and dentate gyrus areas in the coronal portion; The black box denotes the cerebellar cortex; The orange box denotes dentate gyrus areas in the sagittal posterior.

Maynard *et al.* (23) manually annotated the cortical layers (L1–L6) and white matter (WM) of 12 slides of the dorsolateral prefrontal cortex (DLPFC) by gene marker and cytoarchitecture (Figure 2A), which is a publicly available 10 × Visium ST benchmarking dataset. To evaluate the performance of DeepST to identify spatial domains, we compared DeepST with existing state-of-the-art methods on the above-benchmarking dataset. Specifically, DeepST was compared with two non-spatial algorithms (K-means and Seurat (24)) and four recently published spatial clustering algorithms (stLearn (6), SpaGCN (7), SEDR (8), and BayesSpace (5)), and the results demonstrated that the spatial domains identified by DeepST were consistent with the manual annotation of DLPFC and the definition of cortical stratification in neuroscience.

By exhaustive comparison of these methods, we found that the four spatial algorithms leveraging spatial information performed better than the two non-spatial clustering algorithms, which showed that spatial information was needed to correctly identify spatial domains. Strikingly, our proposed DeepST performed better than existing state-of-the-art methods (Figure 2C and Supplementary Figures 2–12). For the boundary division of layers, the ARI of DeepST was 0.515+/0.011, which was substantially higher than that of the current best method (BayesSpace, ARI = 0.463+/0.012; Wilcoxon test, P value = 0.007). DeepST obtained the best clustering accuracy in slide 151671 (ARI = 0.798; Supplementary Figure 8). In slide 151673 (3639 locations and 33 538 genes), DeepST and BayesSpace successfully delineated the L1 and L2 cortical layers, which have never been detected by any other method (Figure 2B). The UMAP and the PAGA (25) (the partition-based graph abstraction) results of DeepST indicated that the various cortical layers were well organized from L1 to L6 and WM, better than the result of Seurat (Figure 2D).

Next, we further evaluated the effectiveness of DeepST in identifying spatial domains in a 10 × Visium dataset of mouse brain tissue, and compared the spatial domains identified by DeepST with the Allen Mouse Brain Atlas (26) brain anatomical reference annotations. DeepST clearly detected the cornu ammonis and dentate gyrus sections of the hippocampal region in the mouse brain (Figure 2F and Supplementary Figure 13B), as well as the cerebellar cortex and the dorsal gyrus (Figure 2F and Supplementary Figure 13A, C) regions in the sagittal posterior, which is consistent with the reference annotations (26). When the number of spatial domains is not *a priori*, DeepST adaptively calculates the optimal clustering resolution (see MATERIALS AND METHODS for details), and obtains higher SC (Figure 2E, SC = 0.143) and lower DB (DB = 1.658) values in mouse brain posterior data. In determining the same number of spatial domains, DeepST also demonstrates its exceptional capacity to identify spatial domains (Figure 2E and Supplementary Figure 13). DeepST and BayesSpace show stronger regional continuity and fewer noise points (Supplementary Figure 13). In terms of performance comparison of algorithms, DeepST processes about 4000 spots and 30 000 genes of spatial data, which takes about 7 min (running on GPU) and about 6G memory, whereas BayesSpace requires about four times longer than DeepST and higher memory usage (Supplementary Figure 1).

### Systematic parameter optimization and integration of DeepST

**Figure 3.**
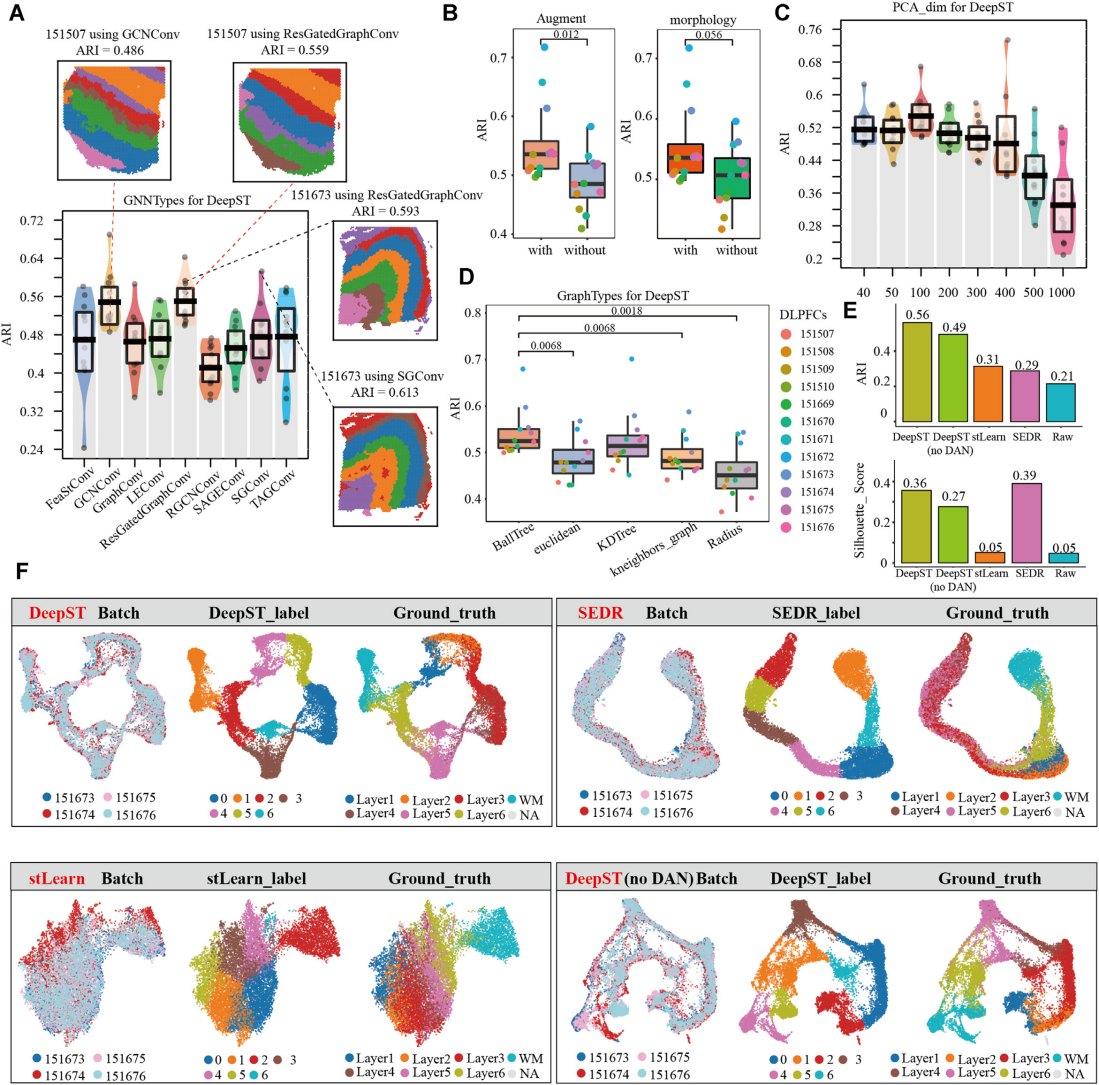
Systematic parameter optimization and integration of DeepST. (**A**) The ARI pirate graph of nine GNN types, each of which was evaluated on 12 DLPFC slides, respectively. Spatial domain distributions of slides 151673 and 151507 with various networks (SGCConv, ResGatedGraphConv and GCNConv) are displayed, respectively. (**B**) ARI boxplots of whether spatial data augmentation is used and whether integrating morphological information in DeepST are shown. (**C**) The ARI pirate graph of reduced dimensions of ST data in DeepST. (**D**) ARI boxplots comparing five methods for constructing adjacency matrices in DeepST. (**E**) Histograms of ARI and Silhouette Coefficients (SC) score for four slides (including 151573, 151574, 151575 and 151576) utilizing spatial algorithms, including DeepST, DeepST (without DAN), SEDR and stLearn. (**F**) UMAP plots of spatial integrated algorithms. They represent batches, recognition spatial domains, and ground truth labels, respectively.

We systematically evaluate DeepST hyperparameters on DLPFC slides. First, we ran nine GNN types (including GCNConv (27), RGCNConv (28), etc.) on 12 DLPFCs and calculated their ARI values (Figure 3A), respectively. GCNConv and ResGatedGraphConv (29) obtain higher ARI values and better model robustness, relatively. The same slide (151673 and 151507) exhibits distinct hierarchical distributions under different network architectures (Figure 3A). The integration of morphological features is what differentiates DeepST from other spatial algorithms. We ran DeepST with or without spatial data augmentation (Figure 3B, *P* value = 0.012), with or without morphological information (Figure 3B, *P* value = 0.056), and with varied augment weights (Supplementary Figure 14A). Spatial data augmentation and rational utilization of morphological image features can significantly improve DeepST performance. Additionally, we evaluated the effect of multiple constructing adjacency matrix methods and dimensionality reduction changes on DeepST performance. There are differences in constructing graph matrix, but their ARI values are not all significant (Figure 3D). The dimension of the data affects the running time and memory usage of the algorithm, and DeepST exhibits relatively stable model performance in 40–300 dimensions (Figure 3C). Some of the remaining parameters are tested (Supplementary Figure 14A, B), including prior knowledge, training epochs, neighbors, etc. Overall, feature embeddings of DeepST exhibit considerable robustness to parameter settings and data processing.

With the widespread application of spatial sequencing technologies, numerous volumes of spatially omics data are being produced. However, it is difficult to compare and integrate multiple datasets from various protocols and technologies. Up-to-date methods (BayesSpace and SpaGCN) are unable to integrate ST datasets from different batches simultaneously. DeepST learns joint embeddings across multiple batches and maps them into a shared latent space, and DAN realizes efficient fusion of multi-batches, thereby reducing technological effect while maintaining biological differences. On four DLPFC slides (151673, 151674, 151675 and 151676), we compared integration performance of other spatial algorithms. DeepST achieves excellent levels of integration between slides, and maximizes the retention of biological content (Figure 3E and F, ARI = 0.56, SC = 0.36; Supplementary Figure 15A and B, ARI = 0.61, SC = 0.38). Interestingly, spatial domain recognition for only one slide (Supplementary Figure 10; such as 151674 ARI = 0.470) is inferior to the result of integrating multiple slides by DeepST (Supplementary Figure 14D; 151674 ARI = 0.588). Furthermore, L1 and L2 layers are clearly delineated, which most spatial algorithms cannot do (Supplementary Figures 10–12 and Supplementary Figure 14D). SEDR can effectively integrate multi-slides and produce higher silhouette coefficient scores (Figure 3E, SC = 0.39), but it lacks inter-layer distinctions, and multi-batches are clustered into the same layered structure (Figure 3F), resulting in a lower ARI (Figure 3E, ARI = 0.29). However, silhouette coefficient can still measure the tightness of multiple batches and the degree of separation between clusters, which can be used as another additional indicator in addition to ARI. When there are no ground truths in ST data, it is also a useful metric for evaluating the clustering performance of spatial algorithms.

At the same time, there is a slight offset between the integrated slides without DAN (Figure 3F). However, when integrating slides with significant batch effects (151507, 151672 and 151673 of DLPFCs), DeepST without DAN has poor batch mixing (Supplementary Figure 15A and B, ARI = 0.24). We performed differential expression analysis on the integrated slides (Supplementary Figure 14C). The differential expression of *MBP* in domain 6 (WM), *PCP4* in domain 1 (L5), and *ENC1* and *ENC2* in domain 3 (L3) keep consistent with previously published results (23,30) (Supplementary Figure 15C–E). These results indicate that DeepST can effectively integrate ST data from multiple batches and different platforms (Figure 5G) while retaining maximal biological content.

### DeepST can dissect spatial domains from cancer tissue at a finer level

**Figure 4.**
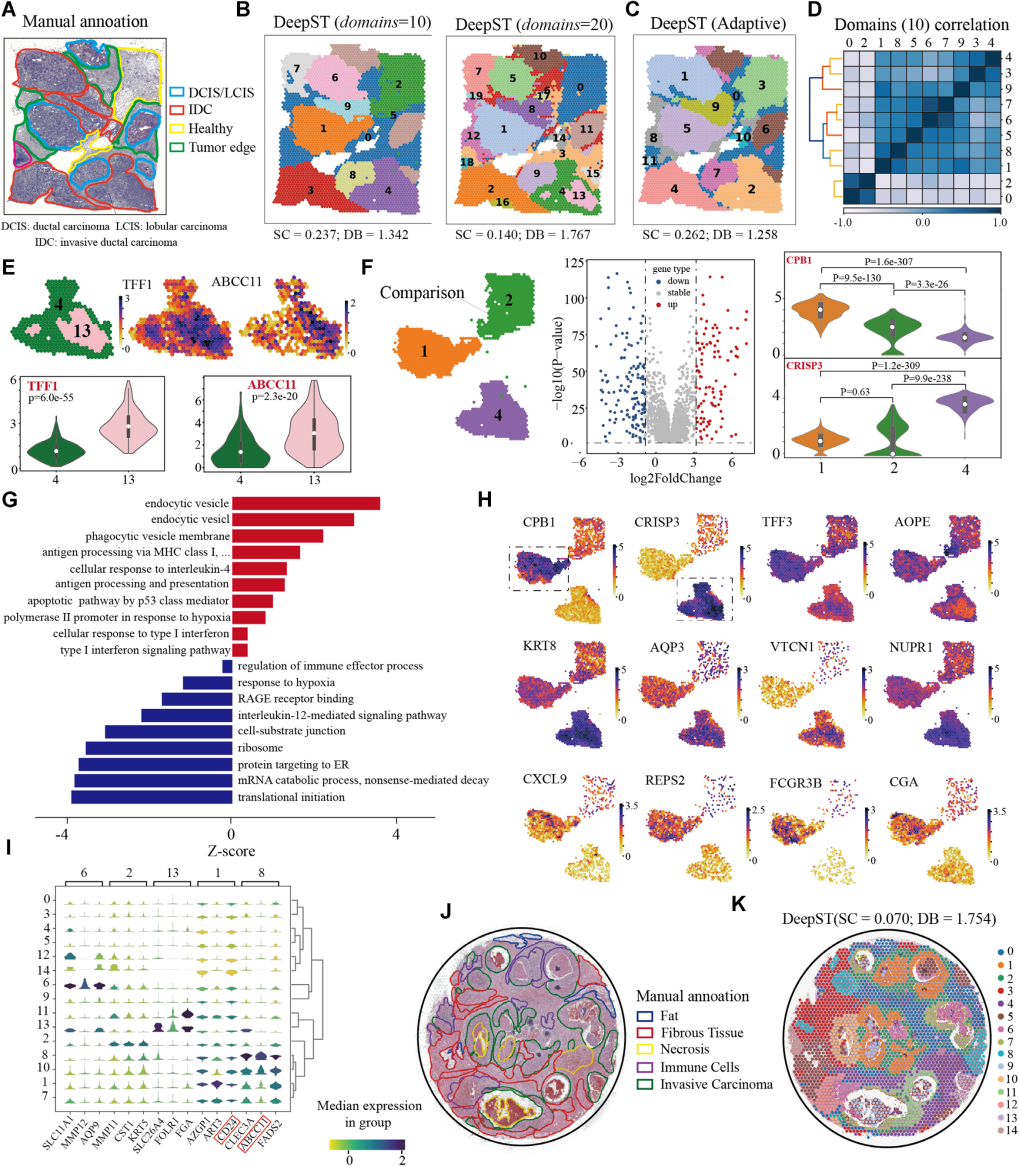
DeepST can dissect spatial domains from cancer tissue at a finer level. (**A**) Visium spatial transcriptomics data of a breast cancer sample annotated by pathologists. IDC, invasive ductal carcinoma; DCIS, ductal carcinoma *in situ*; LCIS, lobular carcinoma *in situ*; tumor edge; healthy region. (**B**) Spatial domains identified by DeepST on human breast cancer (Block A, Section 1) with *domains*= 10 and *domains*= 20. (**C**) Spatial domains were identified without *a priori* knowledge by DeepST. (**D**) Heatmap of Pearson correlation coefficient between domains (*domains*= 10). (**E**) Expression of *TFF1* and *ABCC11* with regional annotation on left (top); Violin plots of the two genes(bottom). (**F**) Differential expression analysis among domains 1, 2 and 4. Spatial location of domains 1, 2 and 4 (left); Volcano graph of DEGs between domains 1 and 4 (*domains*= 10) (middle); *CPB1* and *CRISP3* express differentially between domains 1 and 4 (purple) (right). (**G**) Gene ontology enrichment analysis of the DEGs between domains 1 and 4. Red denotes pathway with upregulated genes in domain 1; blue is the opposite. (**H**) Visualizations of the DEGs (|log fold change|$ \ge$2) between domains 1 and 4 with *k*= 10. *T*-test on the means of two independent domains. (**I**) Stacked violin plot show expression of all domains on the top three DEGs of domains 6, 2, 13, 1 and 8. (**J**) H&E of human breast cancer sample annotated by Agoko's telepathology platform. (**K**) Visium spatial transcriptomics with spatial domains generated by DeepST.

To illustrate the generalization power on cancer tissue, we first tested DeepST on public ST data of breast cancer (Invasive Ductal Carcinoma). We found that the obtained domains were highly consistent with the manual annotations (Figure 4A, B). Compared with domains identified by other spatial algorithms, DeepST discovered regions with more regional continuity and less noise (Figure 4B, Supplementary Figure 16A, B). As expected, spatial domains with high heterogeneity, namely tumour regions, are getting finer as parameters *domain* get bigger (Figure 4B, right). Meanwhile, regions with low heterogeneity, such as healthy regions, still kept consistent regardless of clustering resolution, indicating good robustness of DeepST (Figure 4C, without *a priori* knowledge). At *k* = 20, the increased resolution allowed for more detailed intra-tumour heterogeneity, such as domains 4 and 13 (Figure 4B). Strikingly, these two similar domains were also identified by stLearn and BayesSpace (Supplementary Figure 16B). Differentially expressed genes (DEGs) between domain 4 and domain 13 includes *ABCC11*, *ABCC12* and *TFF1*, in which the first two are multidrug resistance genes and the last one is associated with tumour differentiation. (Figure 4E; Supplementary Figure 17C) (31–34).

To investigate the spatial heterogeneity within the tumour, we calculated the Pearson correlation coefficient between domains (*domains = 10*), and discovered significant heterogeneity between domains 0, 2 and the rest (Figure 4D and Supplementary Figure 17A, B). Here, we mainly compared intratumoral transcriptional differences between domain 1 (ductal/lobular carcinoma, DCIS/LCIS) and 4 (invasive ductal carcinoma, IDC) by performing differential expression analysis followed by pathway enrichment analysis. We detected 298 significant DEGs (|log fold change| ≥2; adjusted *P*-value < 0.05) between domain 1 and 4. (Figure 4F–H and Supplementary Figure 17; Supplementary Table 1). In general, we observed *APOC1*, *APOE*, *C1QB* and *NURP1*, which may reflect differential abundance of tumor-associated infiltration of macrophages (TAM) (35). TAM infiltration is known to be associated with poor survival rate in solid tumors, owing to its promotion of tumor angiogenesis and induction of tumor migration, invasion and metastasis (36,37). In domain 1, *CPB1* can significantly distinguish DCIS from other subtypes of breast cancer (38). We observed natural killer cells (*FCGR3B*; Figure 4H) and lymphocytes (*CXCL9*, *VTCN1*; especially stromal CD3 + and CD8 + T cell; Figure 4H) in domain 1, indicating that it may have more immune cell infiltration. In addition, we observed the upregulation of type I interferon signaling pathway (*ADAR*, *BST2*, *IFI27*, *IFITM3* and *ISG15*; Figure 4G and Supplementary Table 2), *CGA* and *BAMBI*, which exert antitumor and anti-metastatic effects (39–42), pointing towards reduced metastatic potential. Domain 1 represented a region where cancer growth was limited by pro-inflammatory immune response. On the other hand, in domain 4, we observed upregulation of *KRT8*, *AQP3*, *KLHDC7B* and *CDH1*, which tend to exhibit stronger tumor progression and metastasis, and high expression of genes *NUPR1* and *DBI* associated with chemotherapy resistance (Figure 4H and Supplementary Figure 17D) (43,44). Interestingly, we found that domain 13, the core area of domain 4, had low lipid metabolism and high hypoxia response, with low expression of *AOPE* in response to hypoxia (Figure 4H). We also compare transcriptional differences between domain 0 (tumor edge) and domain 4 (Supplementary Figure 18; detailed analyzed results in the legend). Together we showed DeepST is capable of identification of finer regions with different biological functions.

We also examined another ST data of human breast cancer (Ductal Carcinoma In Situ), and the result matched the manually annotated areas as well (Figure 4J, K). DeepST domains are more fluent and continuous than other spatial algorithms (Figure 4K and Supplementary Figure 16C; SC = 0.070 and DB = 1.754), which reflects the ability of DeepST processing to finer divide complex tissues. *AZGP1* levels dictate the histologic grade of breast cancer tumours in domain 1 (45), whereas *ART3* and *CD24* are key triple-negative breast cancer indicators (Figure 4I) (46–49). These results show that DeepST can perform a detailed analysis of the tumor spatial transcriptome and discover more heterogeneity within tumours than was found using other methods, thereby providing a theoretical foundation for the development of targeted treatment strategies.

### DeepST works well on various spatial omics data independent of platforms

**Figure 5.**
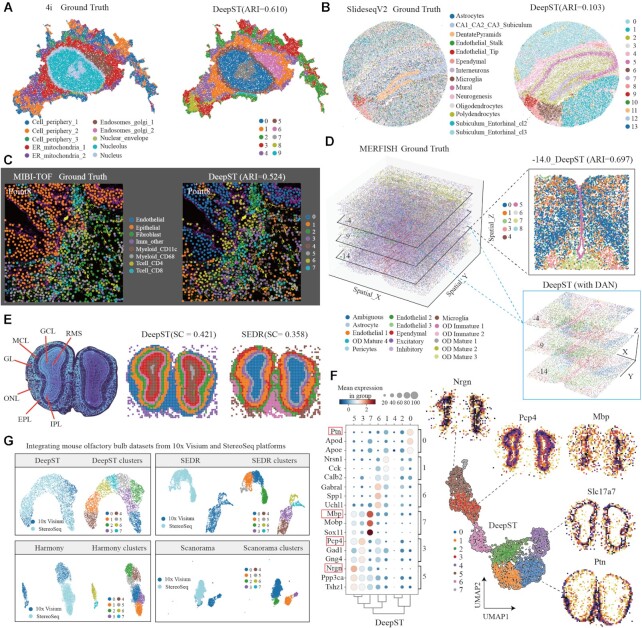
DeepST works on various spatial omics data independent of platforms. (**A**) Visualization of subcellular molecular profiles using 4i (iterative indirect immunofluorescence imaging), plotted in spatial coordinates (left, 25 415 observations/pixels and 43-plex proteins, annotated(9) 10-cell states), and spatial domain identification using DeepST was plotted (right). ER, endoplasmic reticulum. (**B**) Visualization of SlideseqV2 dataset (41 786 sub-cells and 4000 genes) of mouse hippocampus with cell-type annotations (left, annotated by (A). Goeva and Macosko (50)) and spatial domains of DeepST (right). (**C**) Visualization of imaging-based molecular MIBI-TOF(10) dataset (3309 pixels and 36 proteins) with annotations (left, the point 8 section) and spatial domains of DeepST (right, ARI = 0.524) (**D**) Visualization in 3D coordinates in the whole MERFISH(11) dataset (left, annotated) and spatial domains of three consecutive imaging-based molecular slides (lower right, including –4, –9 and –14 layers; using DeepST integrated methods, see Materials and Methods in details). Spatial domain identification using DeepST on –14 imaging slide (top right; ARI = 0.697). (**E**) Nissl-stained coronal section of mouse olfactory bulb (left). Visualization of spatial domains of DeepST (middle) and SEDR (right). RMS, rostral migratory stream; ONL, olfactory nerve layer; IPL, internal plexiform layer; GL, glomerular layer; MCL, mitral cell layer; GCL, granule cell layer; EPL, external plexiform layer. (**F**) Dotplot of the top 3 DEGs of domains 0, 1, 6, 7, 3 and 5 on mouse olfactory bulb by Stereoseq (left). Scatter plot of spatial clustering generated by DeepST (right, including genes *Nrgn*, *Pcp4*, *Mbp*, *Slc17a7* and *Ptn*). (**G**) Visualization of integrated mouse olfactory bulb datasets from two ST technologies (10 × Genomics Visium and Stereoseq) using DeepST, SEDR, Harmony(17) and Scanorama(18), respectively.

Aside from the 10 × Genomics Visium platform, we investigated the generalization ability of DeepST on imaging-based molecular data (MERFISH (11), 4i (9) and MIBI-TOF (10)) and high-resolution ST data (Stereoseq (3) and SlideseqV2 (2)). We first applied DeepST to 4i (iterative indirect immunofluorescence imaging) data that measured 40 protein reads in high-throughput biological samples from the millimeter to nanometer scale (∼270 000 observations/pixels), here only use partial molecular data for spatial domain identification (Supplementary Figure 19A, 25 415 observations). DeepST delineates a more detailed subcellular distribution to the local area than SEDR and stLearn, including various compartments, organelles and cellular structures within each cell (Figure 5A and Supplementary Figure 19A; DeepST ARI = 0.610, stLearn ARI = 0.557 and SEDR ARI = 0.468). Similarly, we applied DeepST to another imaging-based molecular MIBI-TOF data, which imaged 36 labeled antibodies with histochemical staining and endogenous elements (3309 pixels). DeepST reveals partial regional continuity and local element fusion on the four imaging results (Figure 5C and Supplementary Figure 19C–E), which is almost compatible with original annotation (10) (DeepST piont8 ARI = 0.524, SEDR point8 ARI = 0.453).

Following that, we evaluated the performance of DeepST on ST data at approximately single-cell resolution. DeepST shows regional continuity, such as the ‘DentatePyramids’ and ‘Endothelial_Tip’ cell-type annotations, in SlideseqV2 data (41 786 sub-cells and 4000 genes) of mouse hippocampus (Figure 5B). This result is also presented in SEDR algorithm, but it is truncated in the annotation ‘CA1_CA2_CA3_subiculum’ (corresponding to domains 3 and 4; Figure 5B and Supplementary Figure 19B). The algorithm design of DeepST promotes adjacent points to belong a same domain. DeepST presents stronger domain regionality and continuity than ground truth (annotated by A. Goeva and E. Macosko (50)), which may result in low ARI values. Interestingly, DeepST (with DAN) is also capable of processing 3D information, such as MERFISH data from mouse preoptic hypothalamus, but most spatial algorithms may be unable to handle these 3D data owing to repeated spatial coordinates (X and Y). DeepST integrated three consecutive batches of imaging data, clearly deciphering the ‘Ependymal’ and ‘OD Mature’ 3D expression domains (Figure 5D, –14.0 ARI = 0.697), and batch processing provides a clearer 3D molecular structure distribution than single spatial domain identification (Supplementary Figure 20).

We also validated the performance of DeepST on Stereoseq chips (∼11.72 mm^2^) of mouse olfactory bulb. DeepST accurately identified the rostral migratory stream, olfactory nerve layer, internal plexiform layer, glomerular layer, mitral cell layer, granule cell layer and external plexiform layer, matching the known anatomical characteristics (Figure 5E). DeepST exhibits a finer layered distribution and a higher domain silhouette coefficient score than SEDR. We further analyzed DEGs between domains of DeepST, and discovered particular lamellar distribution genes (*Pcp4*, *Slc17a7* and *Sox11*; Figure 5F), which are consistent with previously reported assessments of specific genes in mouse olfactory bulb dataset (2,51). Finally, we integrated mouse olfactory bulb datasets from two ST technologies (10 × Genomics Visium and Stereoseq). From the results, the variability of platform data is substantially higher than that of batches. DeepST demonstrated greater domain fusion than SEDR, Harmony (17) and Scanorama (18) (Figure 5G). At the same time, DeepST preserves a significant amount of biological material (Supplementary Figure 21; *Pcp4* and *Nrgn* in cluster 4, *Ptn* in cluster 3).

## DISCUSSION

In this paper, we propose DeepST, a deep learning framework that integrates spatial location, histology, and gene expression to model spatially embedded representations to identify spatial domains with similar expression and histology. DeepST can not only accurately identify the spatial domain and correct batch effects, but can also be adapted to different ST platforms such as MERFISH, Slide-seq and Stereo-seq. Likewise, DeepST has shown the potential to process other spatial omics data (4i and MIBI-TOF; Figure 5A, C). Being applied to a breast cancer ST dataset, DeepST can dissect spatial domains in cancer tissue at a finer scale.

The model construction of DeepST is flexible. First, it offers a variety of graph neural network types for users to choose, such as RGCNConv and GCNconv. Second, DeepST offers the user multiple preset choices of different ST platforms in the options of parameters. Last, the parameter adjustment of the adjacent graph of DeepST allows users to decide different weights to spatial information, so that spatial domains can be accurately discerned. Besides, DeepST is computationally fast and memory-efficient (Supplementary Figure 1). In terms of model stability, we had conducted multiple independent tests on DeepST, SpaGCN and SEDR, all of which employ unsupervised deep learning methods, and their ARI values showed that all three algorithms were valid (average SEDR = 0.409 ± 0.01, SpaGCN = 0.394 ± 0.03 and DeepST = 0.519 ± 0.01; Supplementary Table 3), but DeepST demonstrated more consistent spatial domains with ground truths (Supplementary Material 1). However, the reproduced SpaGCN and SEDR results had significant performance disparity from the original papers, which might be related to hardware differences and the convergence challenges of unsupervised methods. We can utilize random seeds as constraints for the directionless convergence of unsupervised deep learning methods, but this removes the ability to determine the optimal convergence point. Therefore, the applicability of the model and the necessity for convergence stability must be further considered in our future work.

Rapid advances in ST technology can measure large number of cells through high spatial resolutions, which result in the explosion of ST data. therefore, it is a great challenge to propose new methods to mine the increasing ST data. Computational methods employing GNN require large memory to load the entire graph, which inhibits their application to very large datasets. Therefore, it is an important research topic to optimize memory efficiency through the way of GNN mini-batch, parallel techniques, or even distributed learning systems. The other topic could be an integration of data from spatial omics and snRNA-seq data to further optimize the resolution of ST results and achieve automatic annotation for spatial domains.

In summary, DeepST is a novel promising approach to build an augmented representation of each spot to identify the spatial domain. As more ST data are generated, we expect that DeepST will facilitate the discovery of new principles on cellular organization in a spatial context.

## DATA AVAILABILITY

The code for the DeepST algorithm, and a detailed tutorial are available at https://github.com/JiangBioLab/DeepST.

All datasets used in this paper are published datasets available for download. (1) Human DLPFCs within the spatialLIBD (23) (http://spatial.libd.org/spatialLIBD); (2) Human breast cancer and mouse brain tissue sections datasets (https://support.10xgenomics.com/spatial-gene-expression/datasets); (3) 4i dataset and MIBI-TOF (https://github.com/scverse/squidpy); (4) Stereo-seq dataset for mouse olfactory bulb tissue (https://github.com/BGIResearch/stereopy); and (5) Additional publicly available raw datasets from the spatialDB (52) (https://www.spatialomics.org/SpatialDB/).

## Supplementary Material

gkac901_Supplemental_Files
