# Supplementary material for: DeepST: identifying spatial domains in spatial transcriptomics by deep learning
Source: Nucleic Acids Res. 2022 Oct 17;50(22):e131. doi: 10.1093/nar/gkac901 (PMC9825193; doi:10.1093/nar/gkac901)
Supplement: gkac901_Supplemental_Files [file gkac901_supplemental_files.zip › Supplementary notes and figures.docx]

**Supplementary Notes**

*Comparison of other clustering methods, including spatial algorithms and non-spatial methods.*

1. K-means: The base python *sklearn.cluster import KMeans* function was used with default parameters.
2. stLearn(1): stLearn was applied to the DLPFC datasets as described in their online tutorial (<https://stlearn.readthedocs.io/en/latest/stSME_clustering.html#Human-Brain-dorsolateral-prefrontalcortex-(DLPFC)> ).

**Parameters setting:** To control for the choice of input genes, we ran stLearn’s SME normalization directly on the top 15 PCs we computed from the top 2,000 HVGs. However, in order to compare with the author’s recommended practices, we additionally applied the method to the raw counts of all genes, as shown in the online tutorial.

1. Seurat(2): Seurat (>=4.0) was applied to the DLPFC datasets as described in their online tutorial (<https://satijalab.org/seurat/articles/spatial_vignette.html>).

**Parameters setting:** Raw mRNA counts were preprocessed to remove low-quality genes and transformed to remove technical artifacts and normalize the data. We then ran PCA to extract the top 30 principal components (PCs) and used them to calculate the shared nearest neighbors (SNNs). Then, the Louvain clustering algorithm was used to identify clusters with the SNN networks. We tried clustering at different resolutions to obtain the same number of clusters as the ground truth layers.

1. SpaGCN(3): SpaGCN was applied to the DLPFC datasets as described in their online tutorial (<https://github.com/jianhuupenn/SpaGCN/blob/master/tutorial/tutorial.ipynb>).

**Parameters setting:** We ran these methods with the recommended parameters, including *s*=1, *b*=49, *p*=0.5, *n_clusters*=7 (depending on the number of spatial domains) and *max_epoch*s=200,

and set each one to generate the same number of clusters as the ground truth layers.

1. BayesSpace(4): BayesSpace was applied to the DLPFC datasets as described in their online tutorial (<https://github.com/edward130603/BayesSpace>).

**Parameters setting:** We used the specified settings to run these techniques and configured each one to produce the same number of clusters as the ground truth layers. The function *spatialPreprocess()* log-normalizes the count matrix and performs PCA on the top *n.HVGs* highly variable genes while leaving the top *n.PCs* principal components alone. The *qTune()* and *qPlot()* routines were then used to help choose q, the number of domains to employ in DLPFC analysis. The *spatialCluster()* method clusters the spots and updates the *SingleCellExperiment* with the expected cluster labels.

1. SEDR(5): SEDR was applied to the DLPFC datasets as described in their online tutorial (<https://github.com/JinmiaoChenLab/SEDR/>).

**Parameters setting:** We ran these methods with the recommended parameters and set each one to generate the same number of clusters as the ground truth layers. For spatial domain parameters see: [identify spatial domains](https://github.com/JinmiaoChenLab/SEDR/blob/master/run_SEDR_10x_Genomics_Visium.py). Correcting batch effect parameters see: [correct batch effect](https://github.com/JinmiaoChenLab/SEDR/blob/master/run_UBC_DLPFC_data.py).

1. Harmony(6): By default, we set the following parametersFor the pancreas analysis, we set . We set donors to be the primary covariate () and technology secondary (). In the spatial analysis, we used and .
2. Scanorama(7): We used the assemble function, with precomputed PCs, from the primary github repository (brianhie/scanorama). We set *knn* = 30 and *sigma* = 1, to match the default comparable MNN Correct parameters. All other parameters were kept at default values. We did not use the correct function, as this included both preprocessing and integration of the data. For more equitable comparisons, we tried to use the same preprocessing pipelines for all methods and only compared only the integration steps.
3. DeepST: We give parameter settings of DeepST on all test datasets, and show them in form of each spatial omics data.

**DLPFCs:** *pca_n_comps*=100, *eval_cluster_n*=7 (determined based on ground truth), *distType* ="KDTree", *pre_epochs*=1000, *adjacent_weight*=0.3, *platform*="Visium", *k*=12, *weights*= "weights_matrix_all", *Conv_type*= "GCNConv", *pretrain* = False, *dim_reduction* = True, *priori* = True, *linear_encoder_hidden*= [64,16] and *conv_hidden*=[64,16],

**Mouse brain tissue:** all parameter settings are same as DLPFCs, as above. If the model adaptively selects the number of spatial domains, *priori* =False, otherwise *eval_cluster_n*=15 and *priori* =True.

**Human breast cancer:** all parameter settings are same as DLPFCs, as above, but *eval_cluster_n*=10 or 20.

**MERFISH**(8)**:** *platform* = "merfish", k=6, *pretrain* = True, *priori* = False, *epochs* = 500 *pre_epochs* = 500 and *weights*="weights_matrix_nomd", the remaining parameters are consistent with DLPFCs.

**4i**(9)**:** *platform* = "4i", k=6 and *weights*="weights_matrix_nomd", the remaining parameters are consistent with DLPFCs.

**MIBI-TOF**(10)**:** *platform* = "mibi-tof", k=6 and *weights*="weights_matrix_nomd", the remaining parameters are consistent with DLPFCs.

**SlideseqV2**(11)**:** *platform* = "slideseq", k=6 and *weights*="weights_matrix_nomd", the remaining parameters are consistent with DLPFCs.

**Stereoseq**(12)**:** *platform* = "stereoseq", k=6 and *weights*="weights_matrix_nomd", the remaining parameters are consistent with DLPFCs.

***References***

1. Pham, D., Tan, X., Xu, J., Grice, L.F., Lam, P.Y., Raghubar, A., Vukovic, J., Ruitenberg, M.J. and Nguyen, Q. (2020) stLearn: integrating spatial location, tissue morphology and gene expression to find cell types, cell-cell interactions and spatial trajectories within undissociated tissues. *bioRxiv*, 2020.2005.2031.125658.

2. Stuart, T., Butler, A., Hoffman, P., Hafemeister, C., Papalexi, E., Mauck, W.M., Hao, Y., Stoeckius, M., Smibert, P. and Satija, R. (2019) Comprehensive Integration of Single-Cell Data. *Cell*, **177**.

3. Hu, J., Li, X., Coleman, K., Schroeder, A., Irwin, D.J., Lee, E.B., Shinohara, R.T. and Li, M. (2020) Integrating gene expression, spatial location and histology to identify spatial domains and spatially variable genes by graph convolutional network. *bioRxiv*, 2020.2011.2030.405118.

4. Zhao, E., Stone, M.R., Ren, X., Guenthoer, J., Smythe, K.S., Pulliam, T., Williams, S.R., Uytingco, C.R., Taylor, S.E.B., Nghiem, P. *et al.* (2021) Spatial transcriptomics at subspot resolution with BayesSpace. *Nat Biotechnol*.

5. Fu, H., Xu, H., Chong, K., Li, M., Ang, K.S., Lee, H.K., Ling, J., Chen, A., Shao, L., Liu, L. *et al.* (2021) Unsupervised Spatially Embedded Deep Representation of Spatial Transcriptomics. *bioRxiv*, 2021.2006.2015.448542.

6. Korsunsky, I., Millard, N., Fan, J., Slowikowski, K., Zhang, F., Wei, K., Baglaenko, Y., Brenner, M., Loh, P.-r. and Raychaudhuri, S. (2019) Fast, sensitive and accurate integration of single-cell data with Harmony. *Nat Methods*, **16**, 1289-1296.

7. Hie, B., Bryson, B. and Berger, B. (2019) Efficient integration of heterogeneous single-cell transcriptomes using Scanorama. *Nat Biotechnol*, **37**, 685-691.

8. Moffitt, J.R., Bambah-Mukku, D., Eichhorn, S.W., Vaughn, E., Shekhar, K., Perez, J.D., Rubinstein, N.D., Hao, J., Regev, A., Dulac, C. *et al.* (2018) Molecular, spatial, and functional single-cell profiling of the hypothalamic preoptic region. *Science*, **362**, eaau5324.

9. Gut, G., Herrmann, M.D. and Pelkmans, L. (2018) Multiplexed protein maps link subcellular organization to cellular states. *Science*, **361**, eaar7042.

10. Keren, L., Bosse, M., Thompson, S., Risom, T., Vijayaragavan, K., McCaffrey, E., Marquez, D., Angoshtari, R., Greenwald, N.F., Fienberg, H. *et al.* (2019) MIBI-TOF: A multiplexed imaging platform relates cellular phenotypes and tissue structure. *Science Advances*, **5**, eaax5851.

11. Stickels, R.R., Murray, E., Kumar, P., Li, J., Marshall, J.L., Di Bella, D.J., Arlotta, P., Macosko, E.Z. and Chen, F. (2021) Highly sensitive spatial transcriptomics at near-cellular resolution with Slide-seqV2. *Nat Biotechnol*, **39**, 313-319.

12. Chen, A., Liao, S., Cheng, M., Ma, K., Wu, L., Lai, Y., Qiu, X., Yang, J., Xu, J., Hao, S. *et al.* (2022) Spatiotemporal transcriptomic atlas of mouse organogenesis using DNA nanoball-patterned arrays. *Cell*, **185**, 1777-1792.e1721.


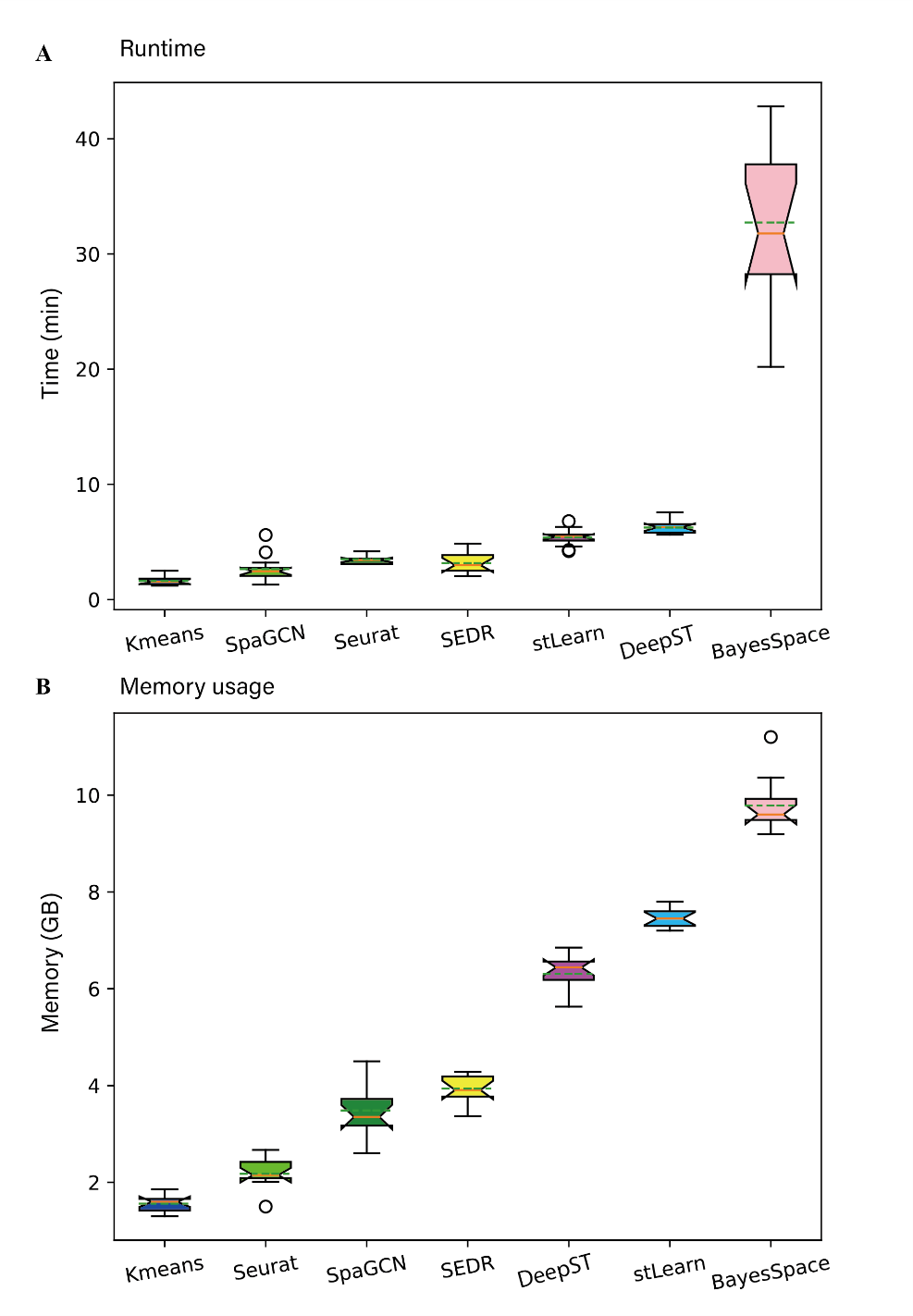


**Supplementary Figure 1.** Runtime and memory benchmarking of the evaluated clustering algorithms.All algorithms completed in under 50 minutes and required less 12GB of memory. (**A**) Runtime of each algorithms (in minutes) on 12 slides from the DLPFC datasets (SpaGCN, SEDR, DeepST and stLearn run on GPU, the rest on CPU). (**B**) Memory consumption of each algorithm on same datasets.


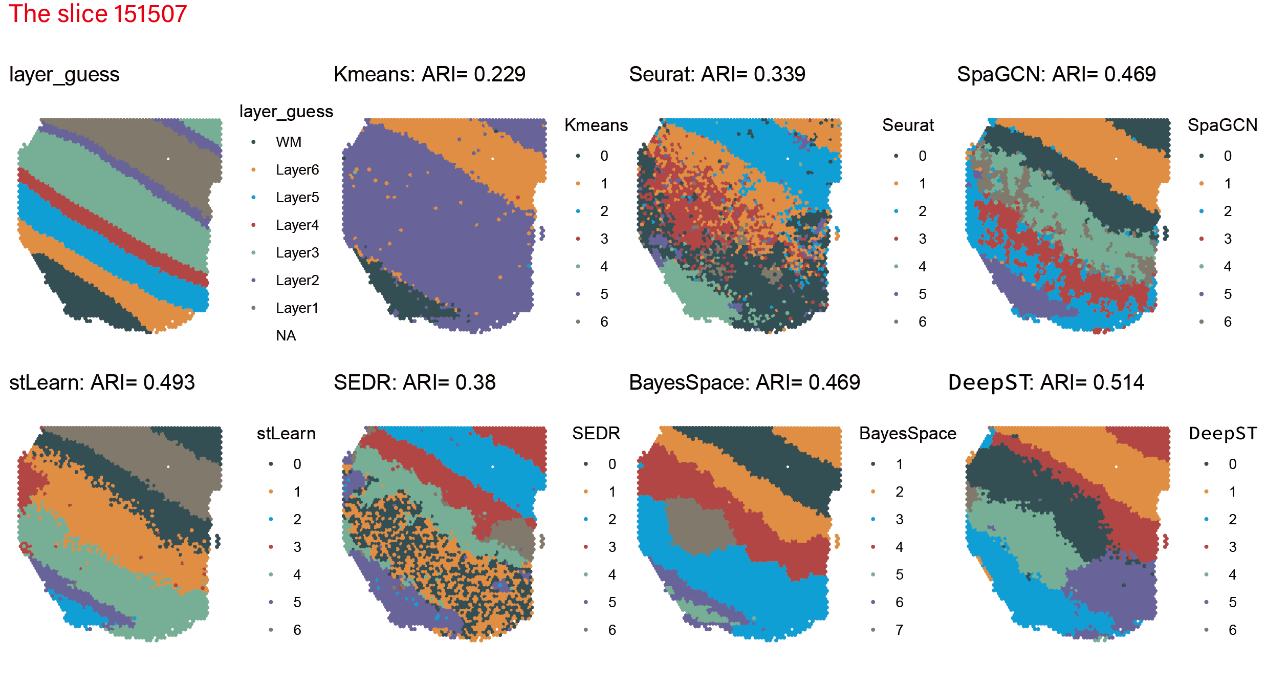


**Supplementary Figure 2.** Performance comparison of all algorithms on 151507 of DLPFCs. Spatial domains recognition results of all methods including spatial algorithms (DeepST, BayesSpace, SpaGCN, stLearn and SEDR) and non-spatial algorithms (Seurat and K-means) for slice 151507. Annotated dorsolateral prefrontal cortex (DLPFC) layer (the first one on left of each figure). Ground truth of spots was mapped on their spatial location, divided into 6 cortical layers (L1-L6) and white matter (WN) layer. DLPFC datasets are accessible (<http://spatial.libd.org/spatialLIBD>).


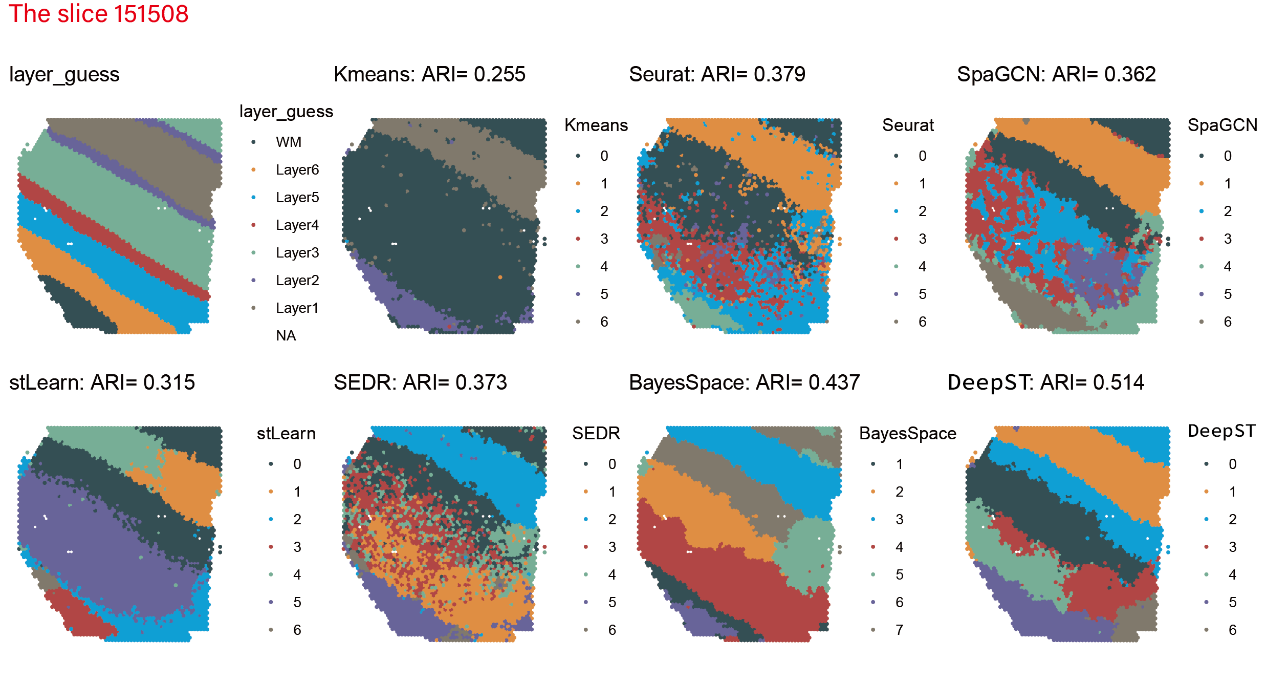


**Supplementary Figure 3.** Performance comparison of all algorithms on slice 151508.Spatial domains recognition results of all methods on slice 151508. Ground truth of spots was mapped on their spatial location, divided into 6 cortical layers (L1-L6) and white matter (WN) layer. DLPFC datasets are accessible (<http://spatial.libd.org/spatialLIBD>).


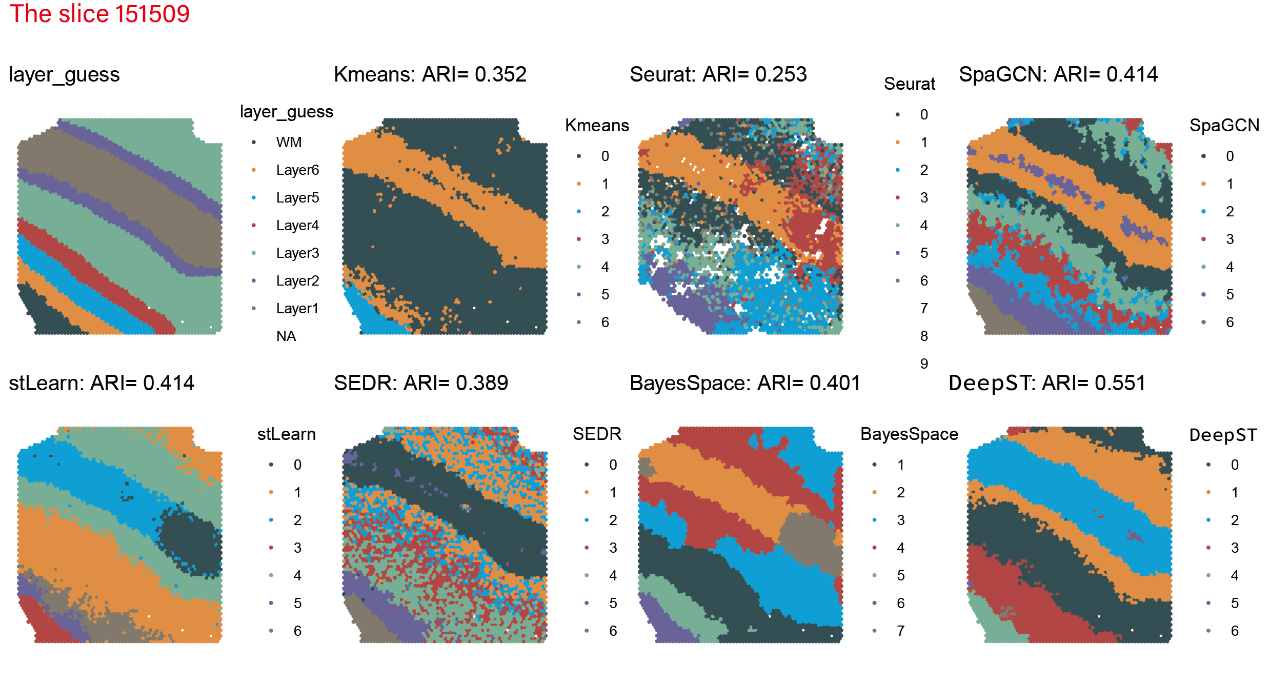


**Supplementary Figure 4.** Performance comparison of all algorithms on slice 151509.Spatial domains recognition results of all methods on slice 151509. Ground truth of spots was mapped on their spatial location, divided into 6 cortical layers (L1-L6) and white matter (WN) layer. DLPFC datasets are accessible (<http://spatial.libd.org/spatialLIBD>).


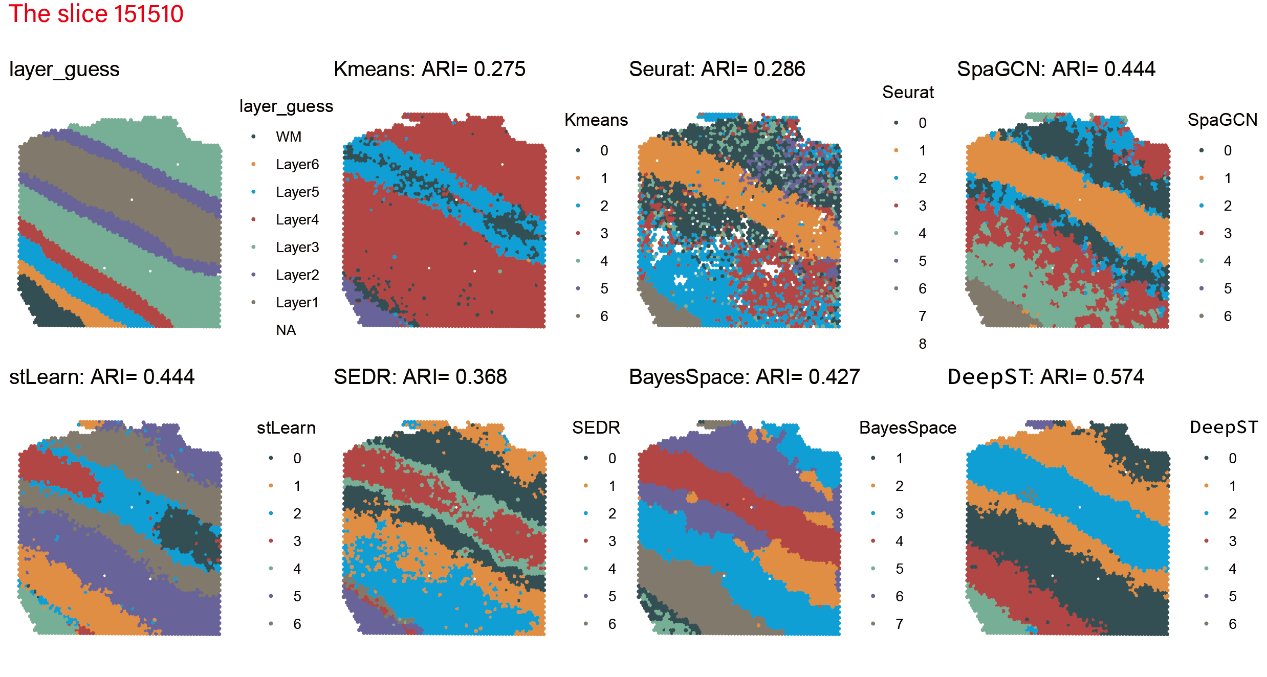


**Supplementary Figure 5.** Performance comparison of all algorithms on slice 151510.Spatial domains recognition results of all methods on slice 151510. Ground truth of spots was mapped on their spatial location, divided into 6 cortical layers (L1-L6) and white matter (WN) layer. DLPFC datasets are accessible (<http://spatial.libd.org/spatialLIBD>).


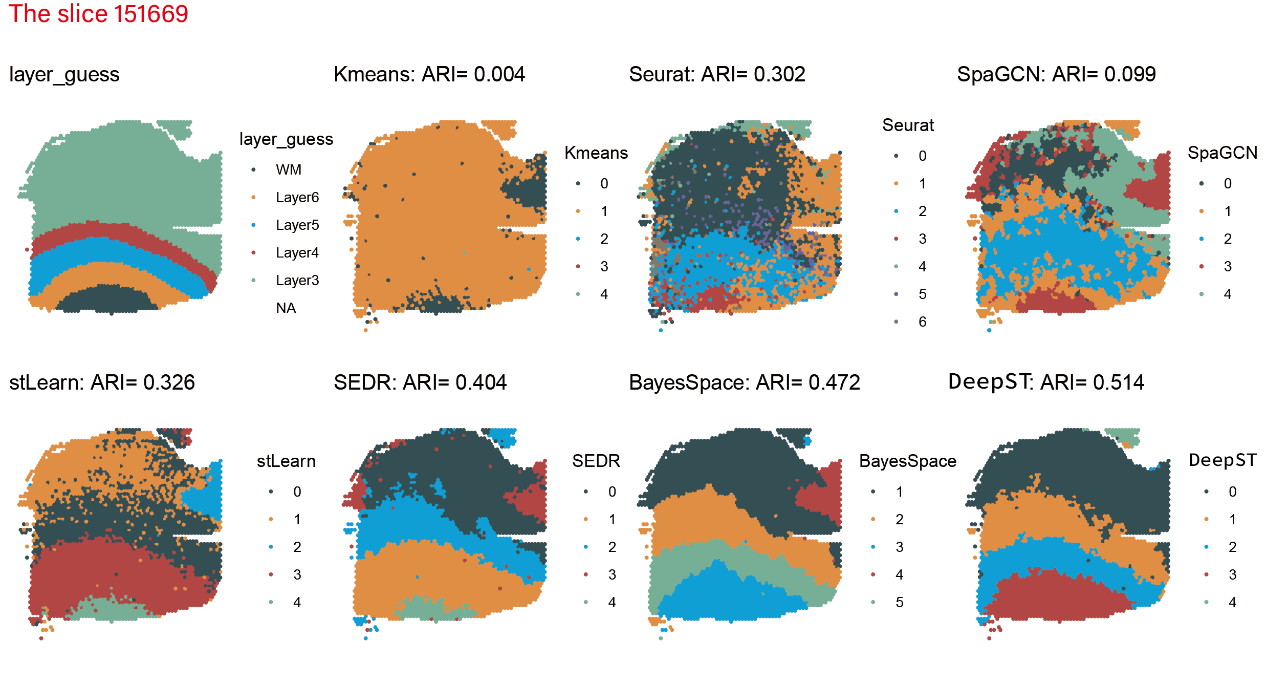


**Supplementary Figure 6.** Performance comparison of all algorithms on slice 151669.Spatial domains recognition results of all methods on slice 151669. Ground truth of spots was mapped on their spatial location, divided into 6 cortical layers (L3-L6) and white matter (WN) layer. DLPFC datasets are accessible (<http://spatial.libd.org/spatialLIBD>).


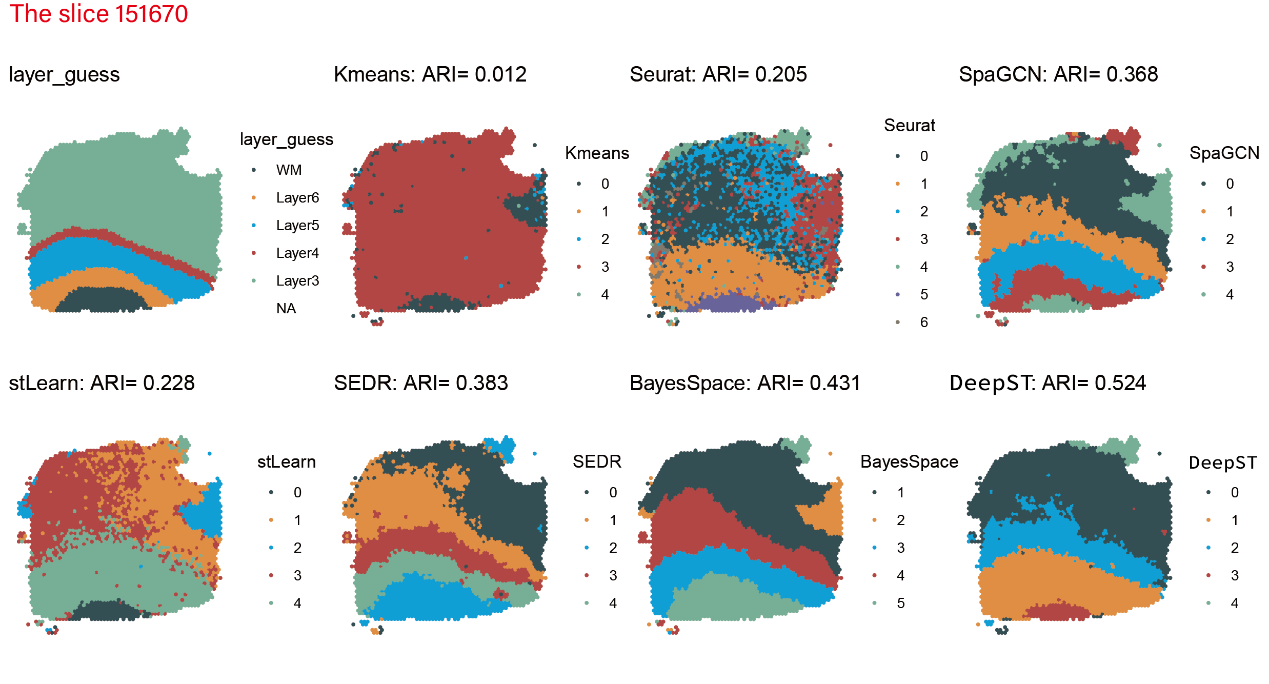


**Supplementary Figure 7.** Performance comparison of all algorithms on slice 151670.Spatial domains recognition results of all methods on slice 151670. Ground truth of spots was mapped on their spatial location, divided into 6 cortical layers (L3-L6) and white matter (WN) layer. DLPFC datasets are accessible (<http://spatial.libd.org/spatialLIBD>).


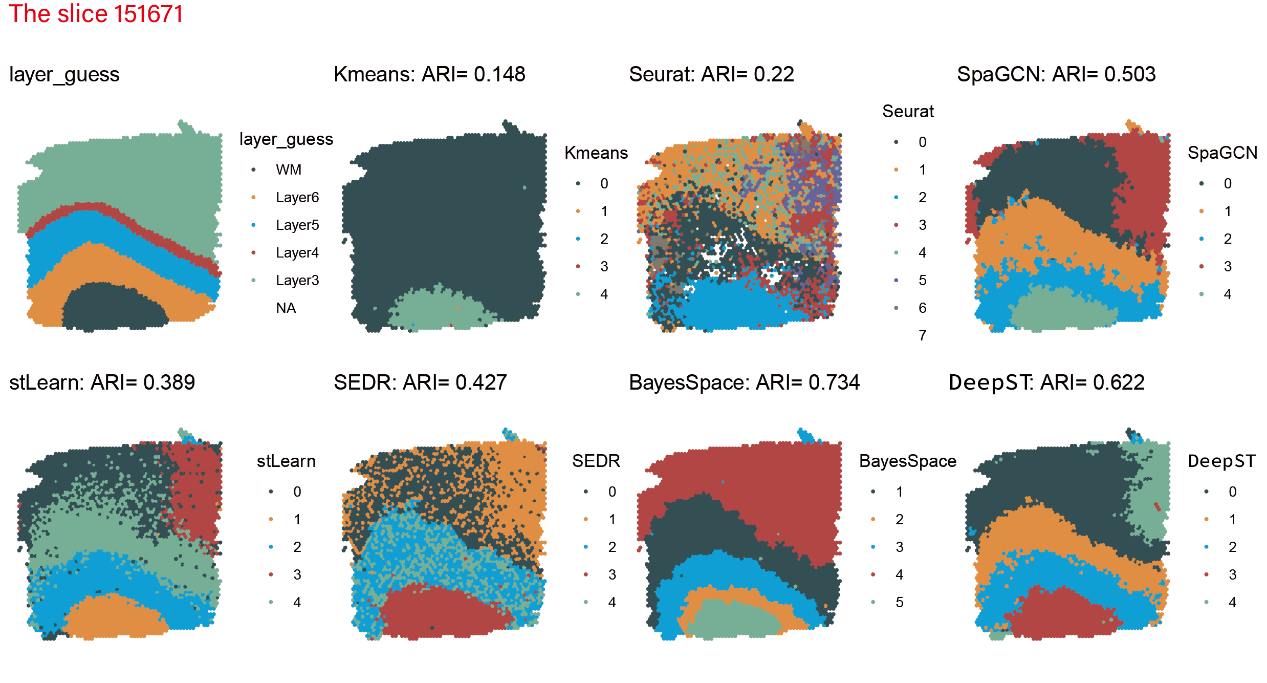


**Supplementary Figure 8.** Performance comparison of all algorithms on slice 151671.Spatial domains recognition results of all methods on slice 151671. Ground truth of spots was mapped on their spatial location, divided into 6 cortical layers (L3-L6) and white matter (WN) layer. DLPFC datasets are accessible (<http://spatial.libd.org/spatialLIBD>).


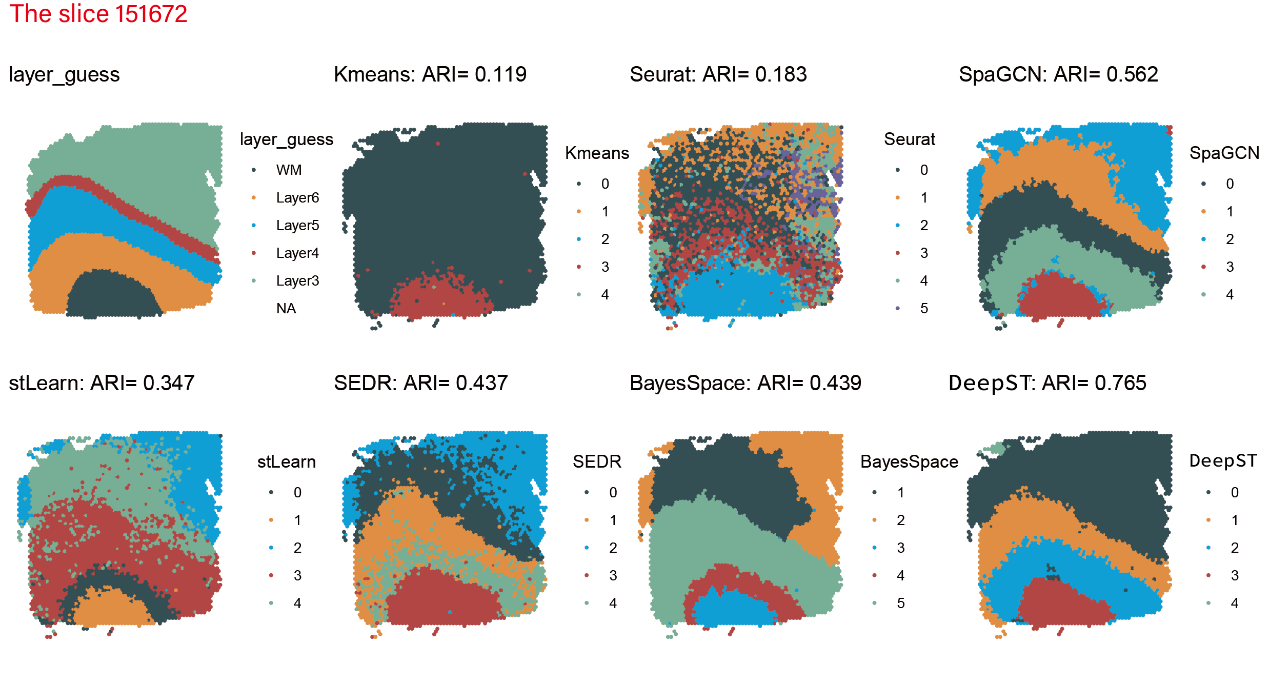


**Supplementary Figure 9.** Performance comparison of all algorithms on slice 151672.Spatial domains recognition results of all methods on slice 151672. Ground truth of spots was mapped on their spatial location, divided into 6 cortical layers (L3-L6) and white matter (WN) layer. DLPFC datasets are accessible (<http://spatial.libd.org/spatialLIBD>).


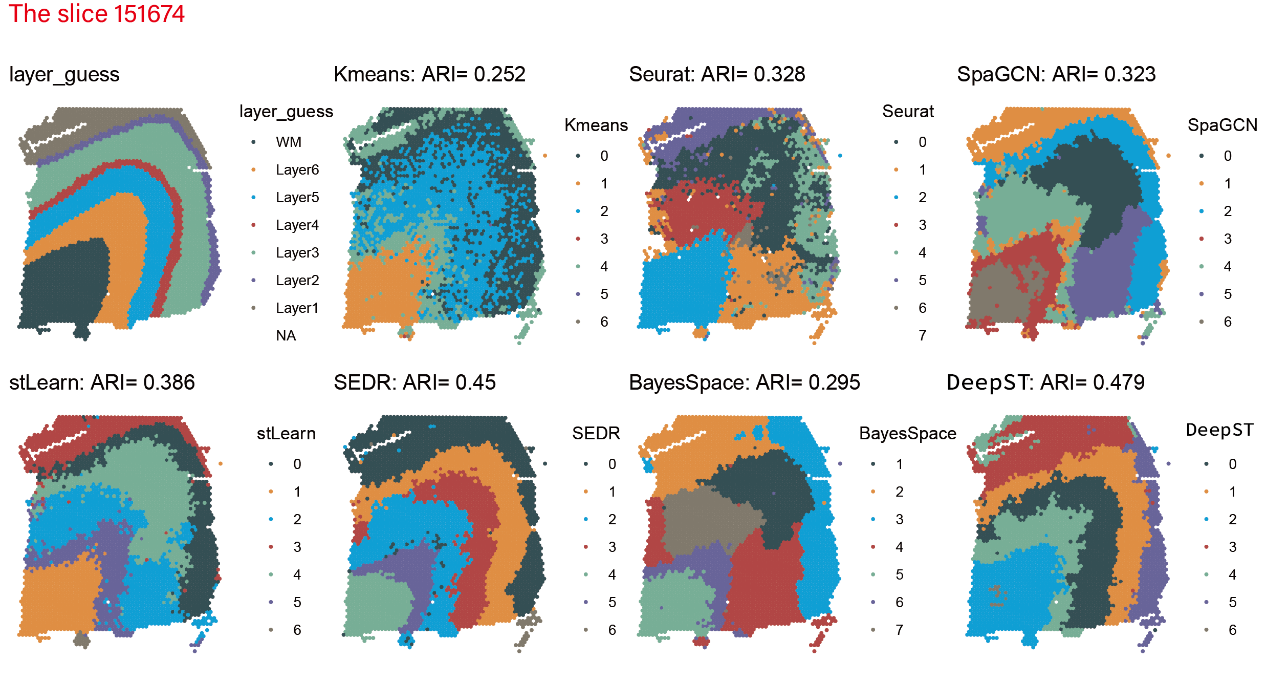


**Supplementary Figure 10.** Performance comparison of all algorithms on slice 151674.Spatial domains recognition results of all methods on slice 151674. Ground truth of spots was mapped on their spatial location, divided into 6 cortical layers (L1-L6) and white matter (WN) layer. DLPFC datasets are accessible (<http://spatial.libd.org/spatialLIBD>).


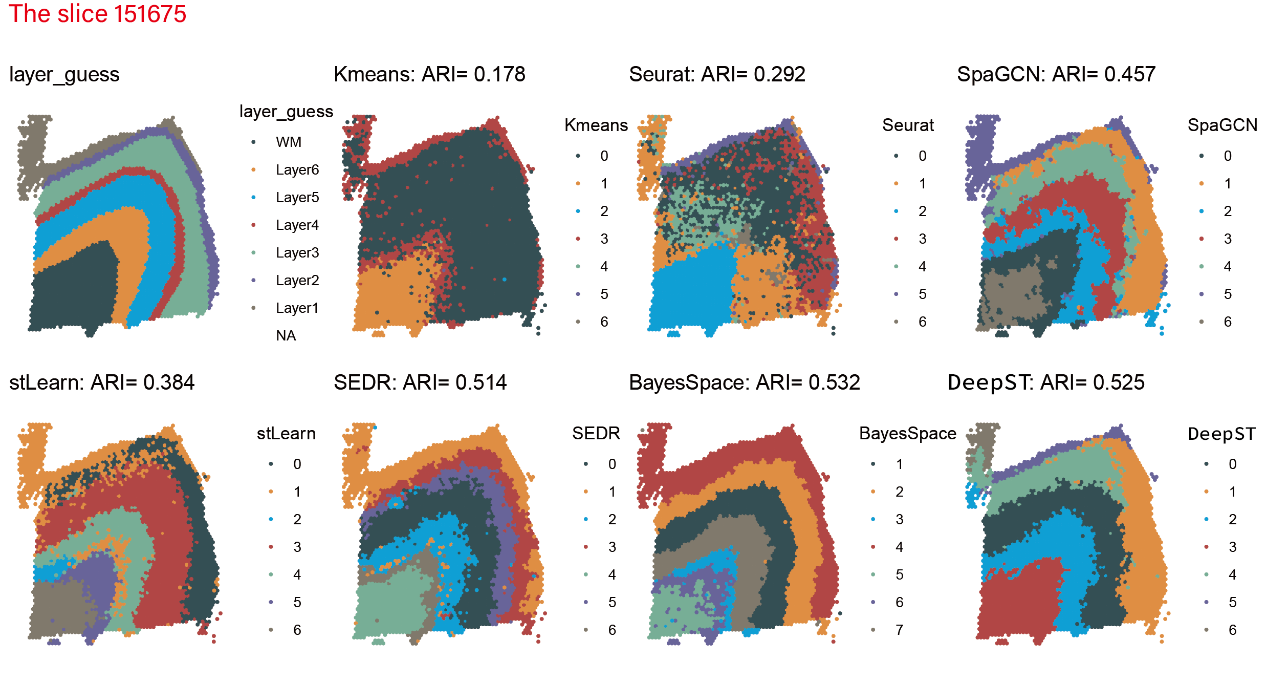


**Supplementary Figure 11.** Performance comparison of all algorithms on slice 151675.Spatial domains recognition results of all methods on slice 151675. Ground truth of spots was mapped on their spatial location, divided into 6 cortical layers (L1-L6) and white matter (WN) layer. DLPFC datasets are accessible (<http://spatial.libd.org/spatialLIBD>).


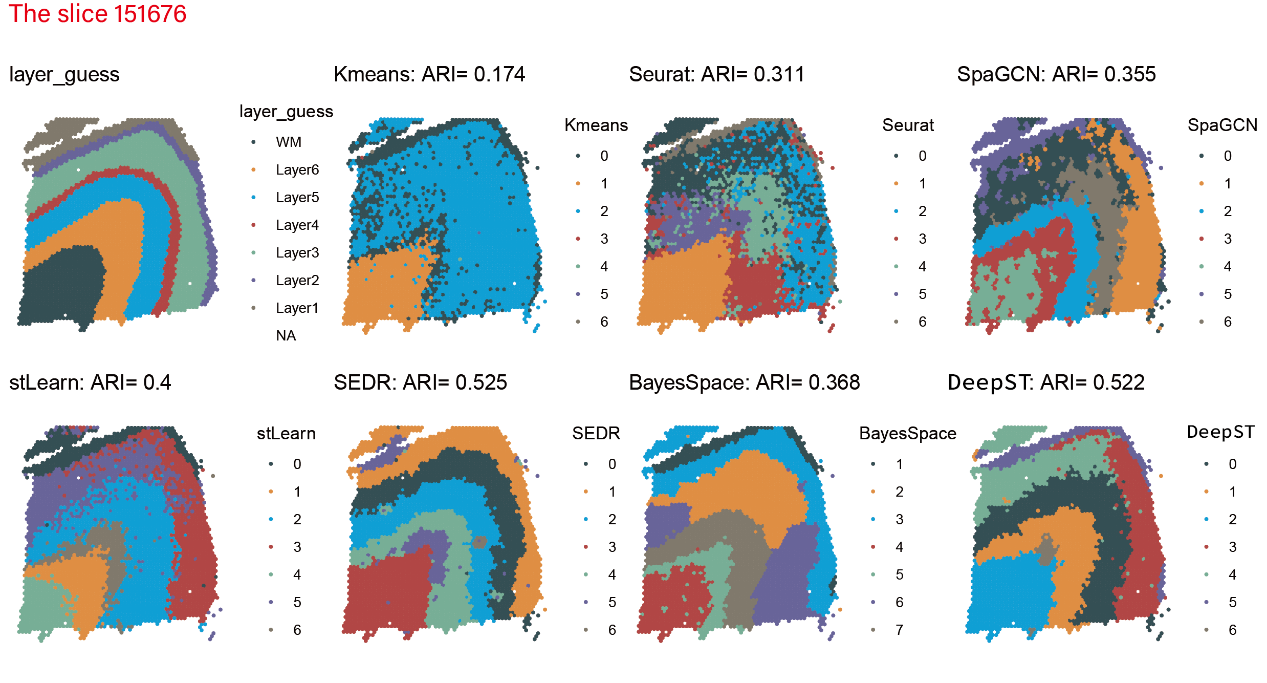


**Supplementary Figure 12.** Performance comparison of all algorithms on slice 151676. Spatial domains recognition results of all methods on slice 151676. Ground truth of spots was mapped on their spatial location, divided into 6 cortical layers (L1-L6) and white matter (WN) layer. DLPFC datasets are accessible (<http://spatial.libd.org/spatialLIBD>).


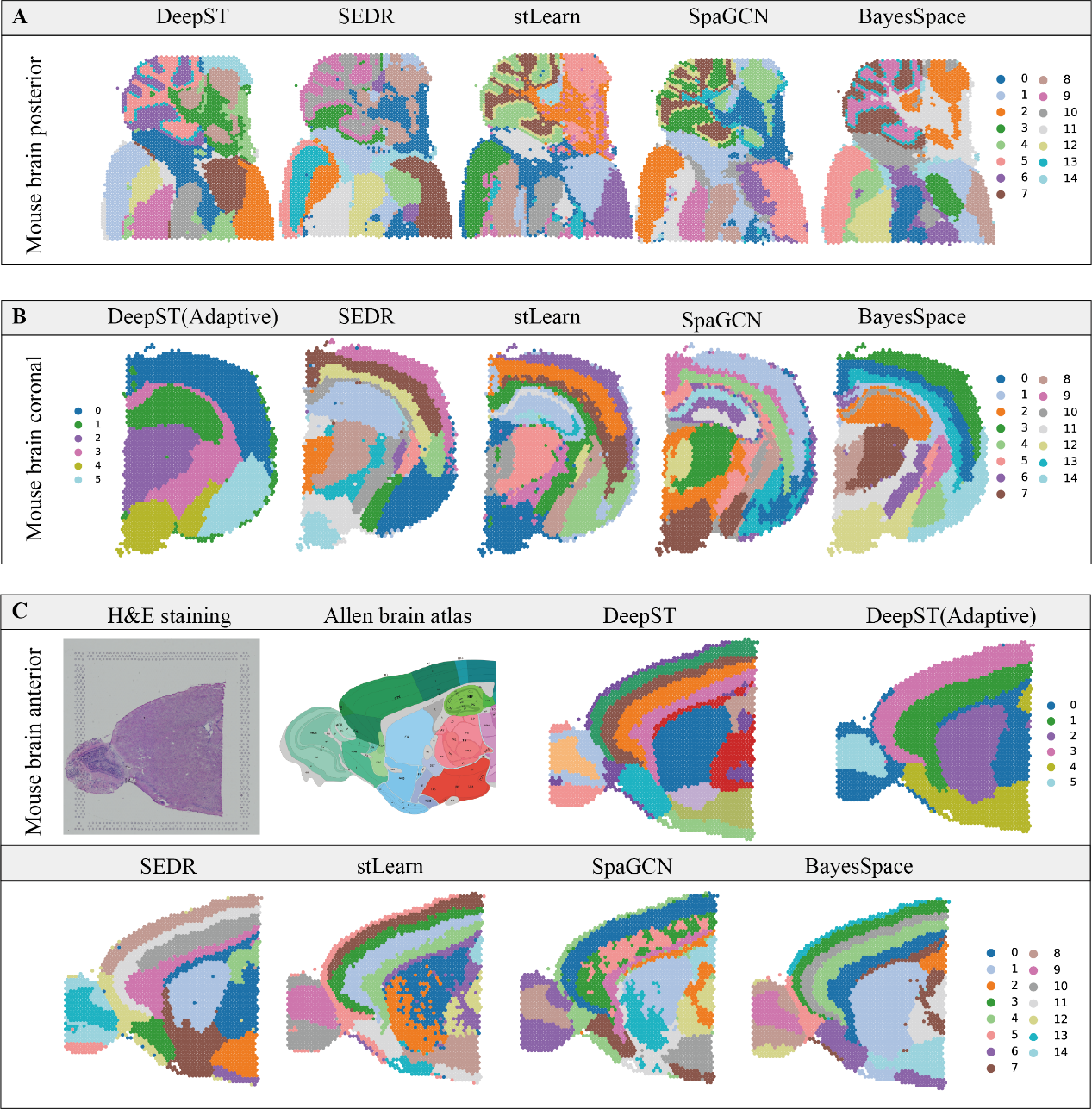


**Supplementary Figure 13.** Spatial domains of mouse brain tissue. (**A**) and (**B**) Spatial domain identification results of five algorithms (DeepST or Adaptive, SEDR, stLearn, SpaGCN and BayesSpace) in mouse brain posterior and coronal, respectively. (**C**) The H&E staining generated from raw data (left), the corresponding anatomical Allen Mouse Brain Atlas (<https://atlas.brain-map.org/>), and spatial domain identification results are shown (including DeepST, DeepST (Adaptive), SEDR, stLearn, SpaGCN and BayesSpace algorithms).


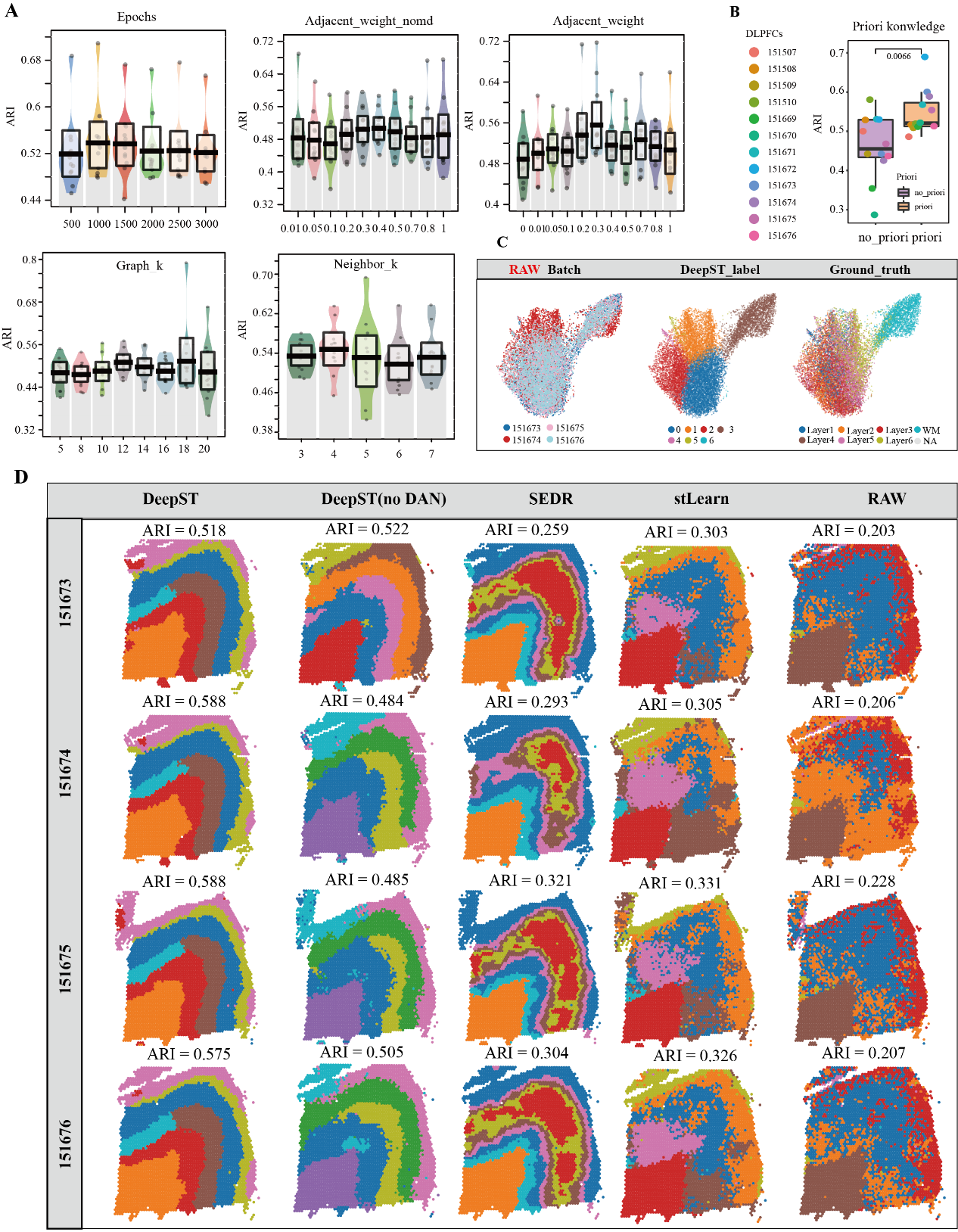


Supplementary Figure 14. Systematic parameter optimization and integration of DeepST. (A) The ARI pirate diagram of DeepST with different parameters on 12 DLPFC slides, including training epochs (left), adjacent weight (middle, without morphological information), adjacent weight (right, with morphological information), the number of adjacent spots in constructing graphs (lower left) and the number of neighbors (lower right). (B) The ARI boxplot of whether using priori knowledge (the number of spatial domains) is shown. (C) The UMAP of raw gene expression integration. (D) Spatial domain recognition graphs of 151673, 151674, 151675 and 151676 slides with different integration algorithms, respectively.


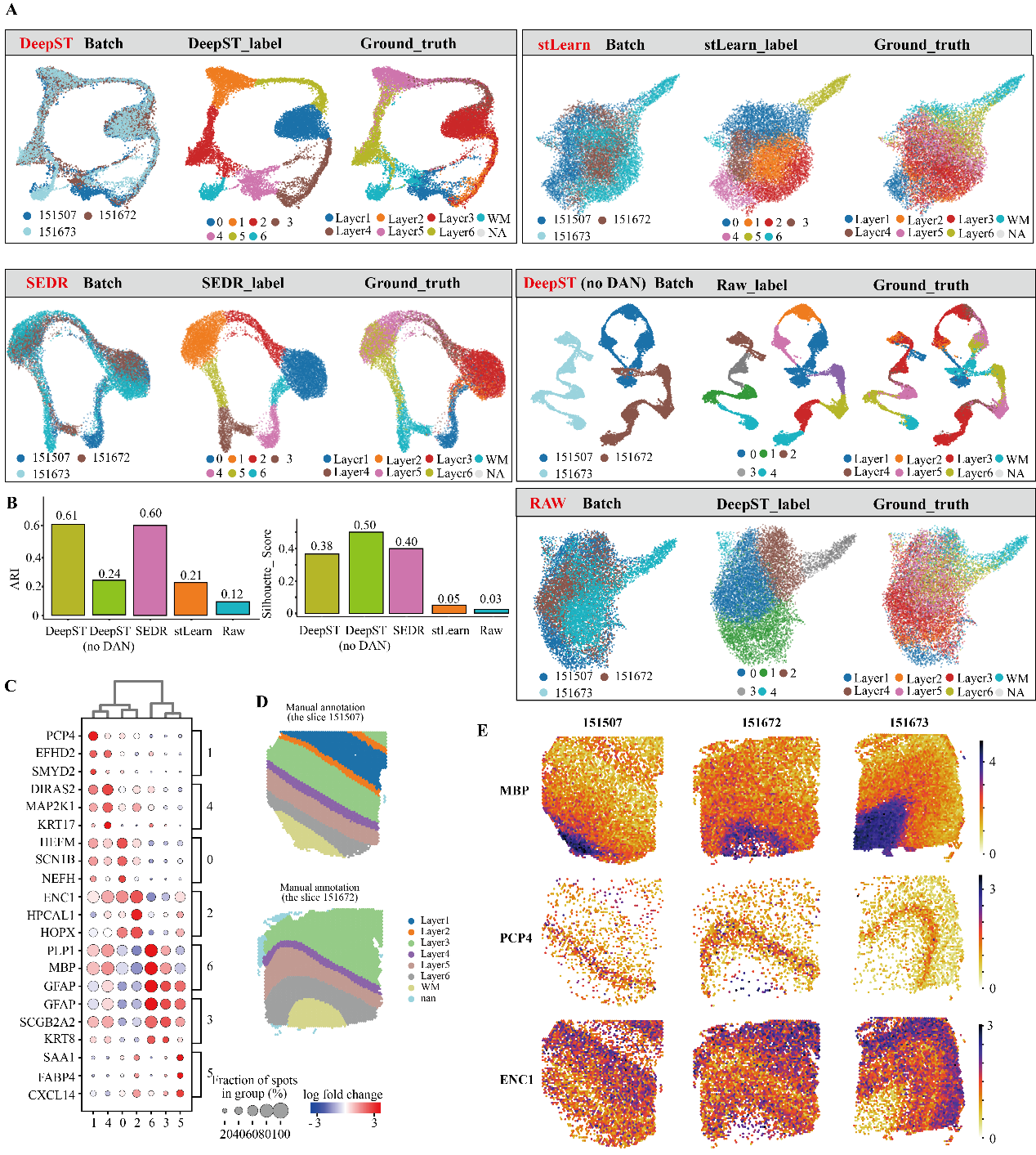


**Supplementary Figure 15.** DeepST corrects batch effects on DLPFC datasets. (**A**) UMAP plots of spatial integrated algorithms, including DeepST, DeepST (without DAN), SEDR and stLearn They show batches, recognition spatial domains, and ground truth labels, respectively. (**B**) Histograms of ARI and Silhouette Coefficients (SC) score for four slides (including 151507, 151572 and 151573) using spatial algorithms. (**C**) A divergent color dotplot of DeepST’s spatial clustering results for the above slides. We plotted log fold changes instead of gene expression. Also, we want to focus on genes that have a log fold change >=2 between groups. (**D**) Annotated DLPFC layers. In slice 151507, the ground truth of spots was mapped on their spatial location, divided into 6 cortical layers (L1-L6), white matter (WM) layer and nan layer. Particularly, the slide 151672 does not contain L1-L2 layers. Annotated layers for slice 151673 can be seen in Figure 2A. (**E**) Visualizations of the corresponding marker genes (MBP, PCP4 and ENC1 genes) for clusters 6, 1 and 2 of slides 151507, 151672 and 151673, respectively.


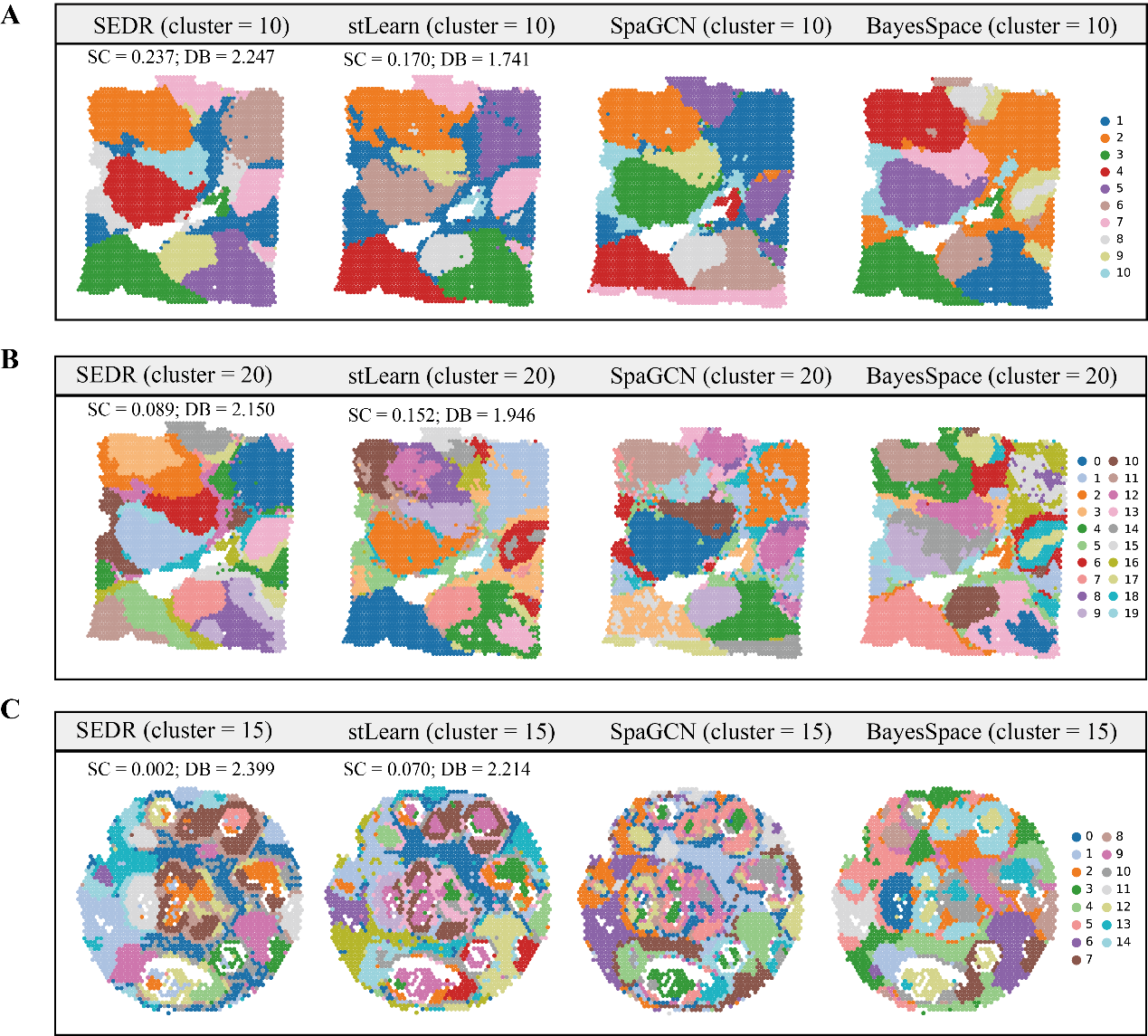


**Supplementary Figure 16.** Spatial domain identification with different spatial algorithms.(**A**) Spatial domains identified on human breast cancer ([Block A Section 1](file:///C:\Users\86130\Desktop\STMAP.docx)) with *k*=10 by four spatial algorithms, including SEDR, stLearn, SpaGCN and BayesSpace. (**B**) Spatial domains identified on human breast cancer ([Block A Section 1](file:///C:\Users\86130\Desktop\STMAP.docx)) with *k*=20 by four spatial algorithms, including SEDR, stLearn, SpaGCN and BayesSpace. (**C**). Visium spatial transcriptomics with spatial domains generated on human breast cancer [(Ductal Carcinoma In Situ)](https://www.10xgenomics.com/resources/datasets/human-breast-cancer-ductal-carcinoma-in-situ-invasive-carcinoma-ffpe-1-standard-1-3-0) data by four spatial algorithms, including SEDR, stLearn, SpaGCN and BayesSpace.


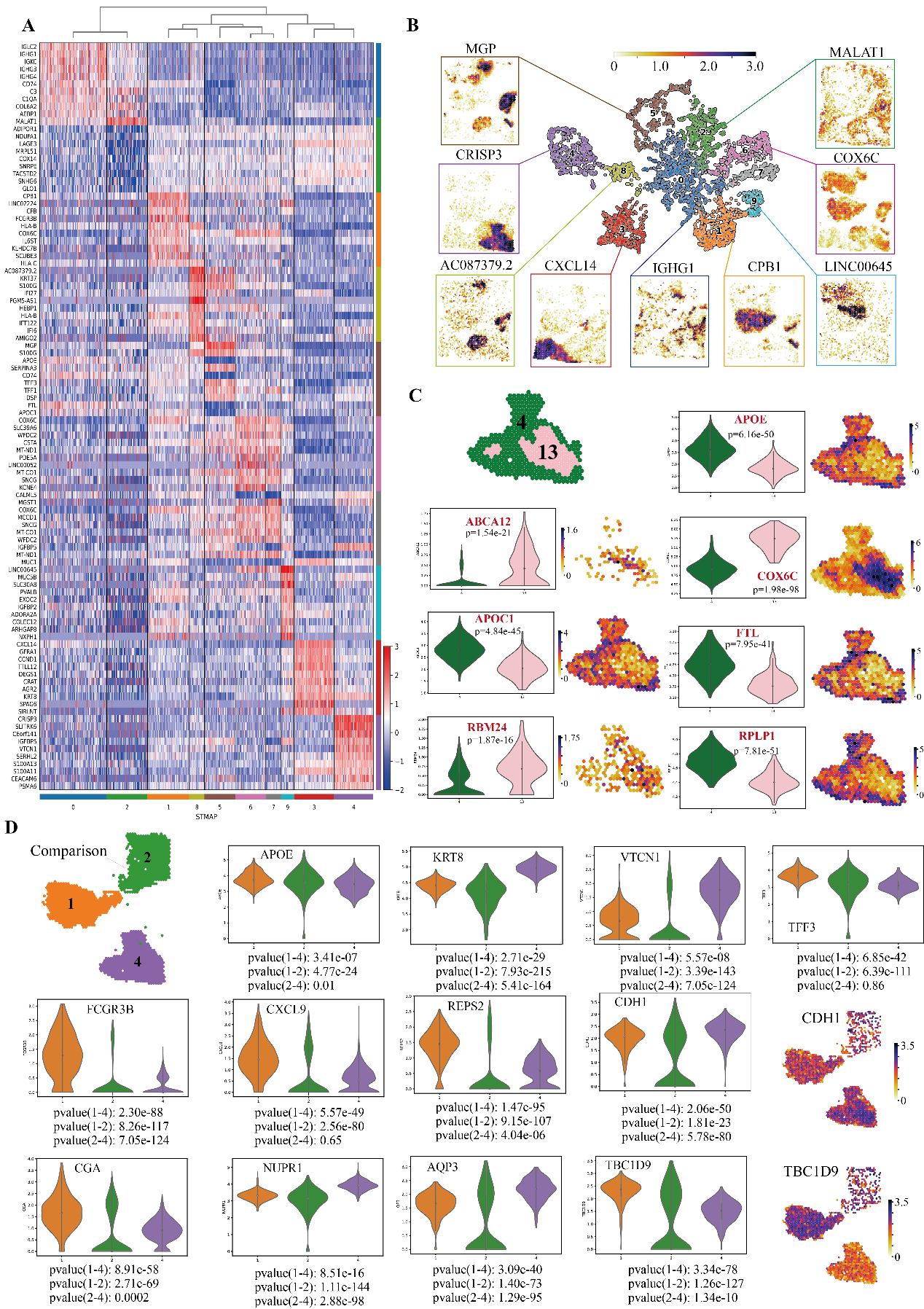


**Supplementary Figure 17.** DEGs in DeepST’s clusters in human breast cancer datasets.(**A**) A heatmap of DEGs on breast cancer dataset, when k=10. We show the top 10 differentially expressed genes (with respect to average log-fold-change) in each DeepST cluster. (**B**) UMAP visualizations and scatter graphs generated (top 1 differentially expressed gene of each domain) using scanpy package. (**C**) Violin plots of differentially expressed genes (e.g., *COX6C*, *ABCA11*, *ABCA12*, where |logFC|>1) between domain 4 and domain 13, when *domains*=20. (**D**) Details of DEGs when *domains=10*. Violin plots of differentially expressed genes and scatter graphs (e.g., *CDH1*, *TBC1D9*, where |logFC|>2). Calculate the T-test for the means of two domains (1 vs 4, 1 vs 2 and 2 vs 4) genes of pvalues.


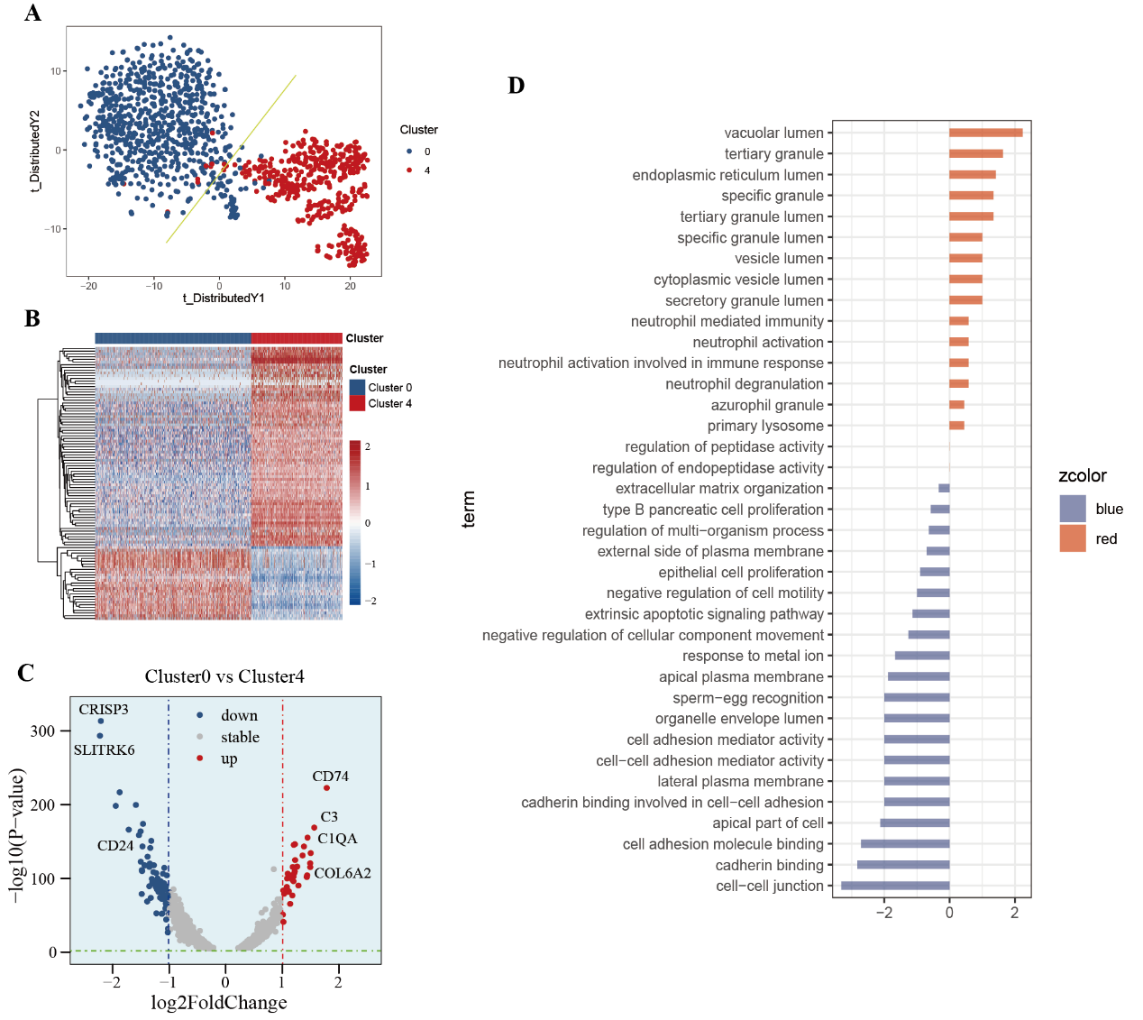


**Supplementary Figure 18.** Differential expression analysis and heterogeneity of domain 0 and domain 4 on human breast dataset. (**A**) The tsne dotplot of domain 0 (non-tumor) vs domain 4 (tumor) when *domains*=10. There is an obvious difference between the two clusters. (**B**) Gene differential expression heatmap of domain 0 vs domain 4 when *domains*=10. It shows that significant differences in gene expression. (**C**) A volcano graph of differential genes (*domains*=10). Red spots represent up-regulated genes, and blue spots represent down-regulated genes in the figure. (**D**) Biological function enrichment analysis. Red bars represent upregulated in domain 0. Overall, domain 0 represented a region with immunosuppressive tumor-promoting microenvironment and high potential for cancer metastasis. Domain 4 represented a region where the pro-inflammatory immune response restricts cancer growth.


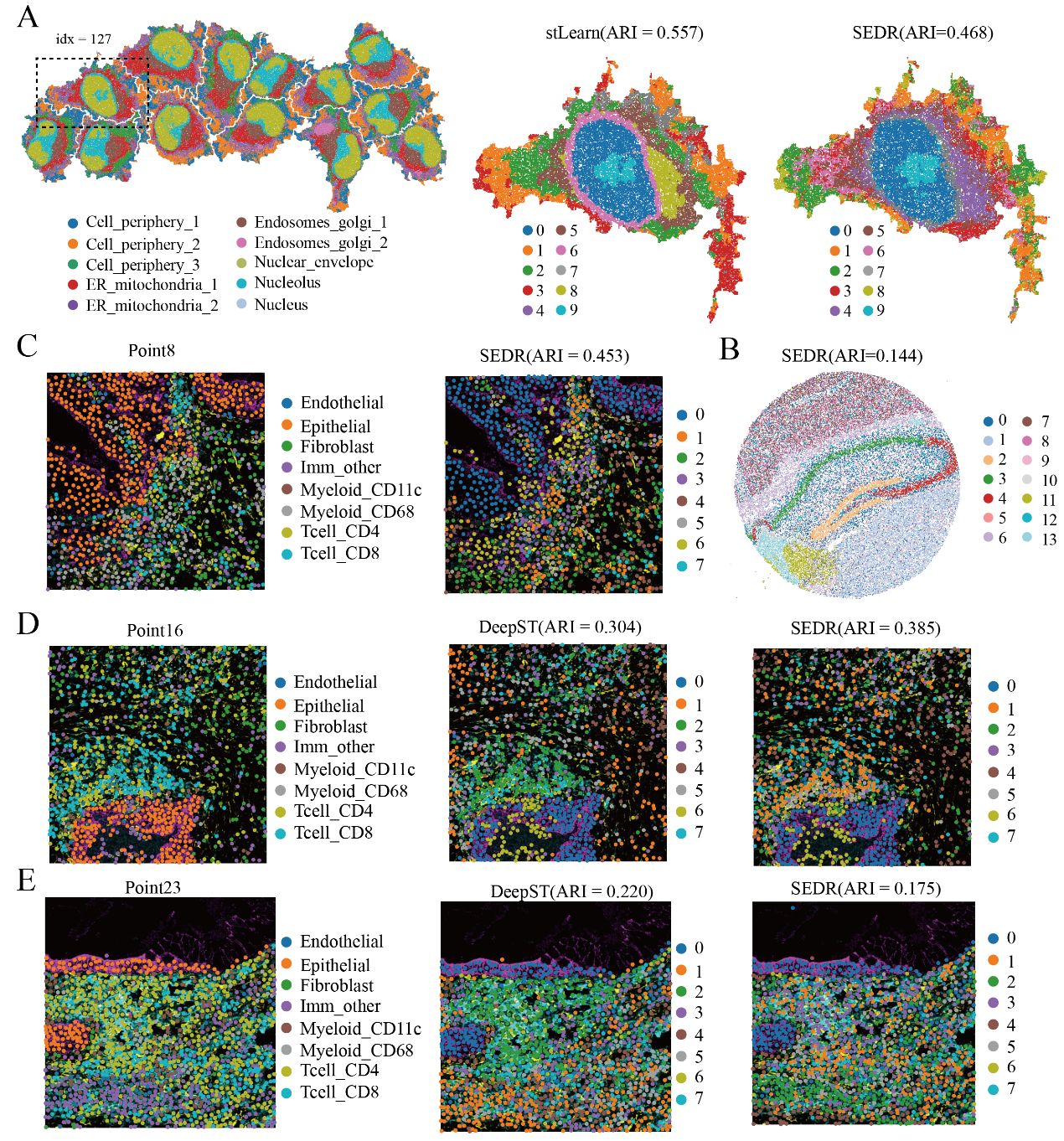


**Supplementary Figure 19.** DeepST works on various spatial omics data independent of platforms. (**A**) Visualization of subcellular molecular profiles using the full 4i (iterative indirect immunofluorescence imaging, left). Spatial domain identification using SEDR (right, only idx = 127) and stLearn (middle) was plotted, respectively. (**B**) Visualization of SlideseqV2 dataset (41,786 sub-cells and 4,000 genes) of spatial domains of SEDR (ARI = 0.144). (**C**), (**D**) and (**E**) Visualization of imaging-based molecular MIBI-TOF dataset (3,309 pixels and 36 proteins) with annotations (left, including point 8, point 16 and point 23) and spatial domains of DeepST (middle) and SEDR (right), respectively.


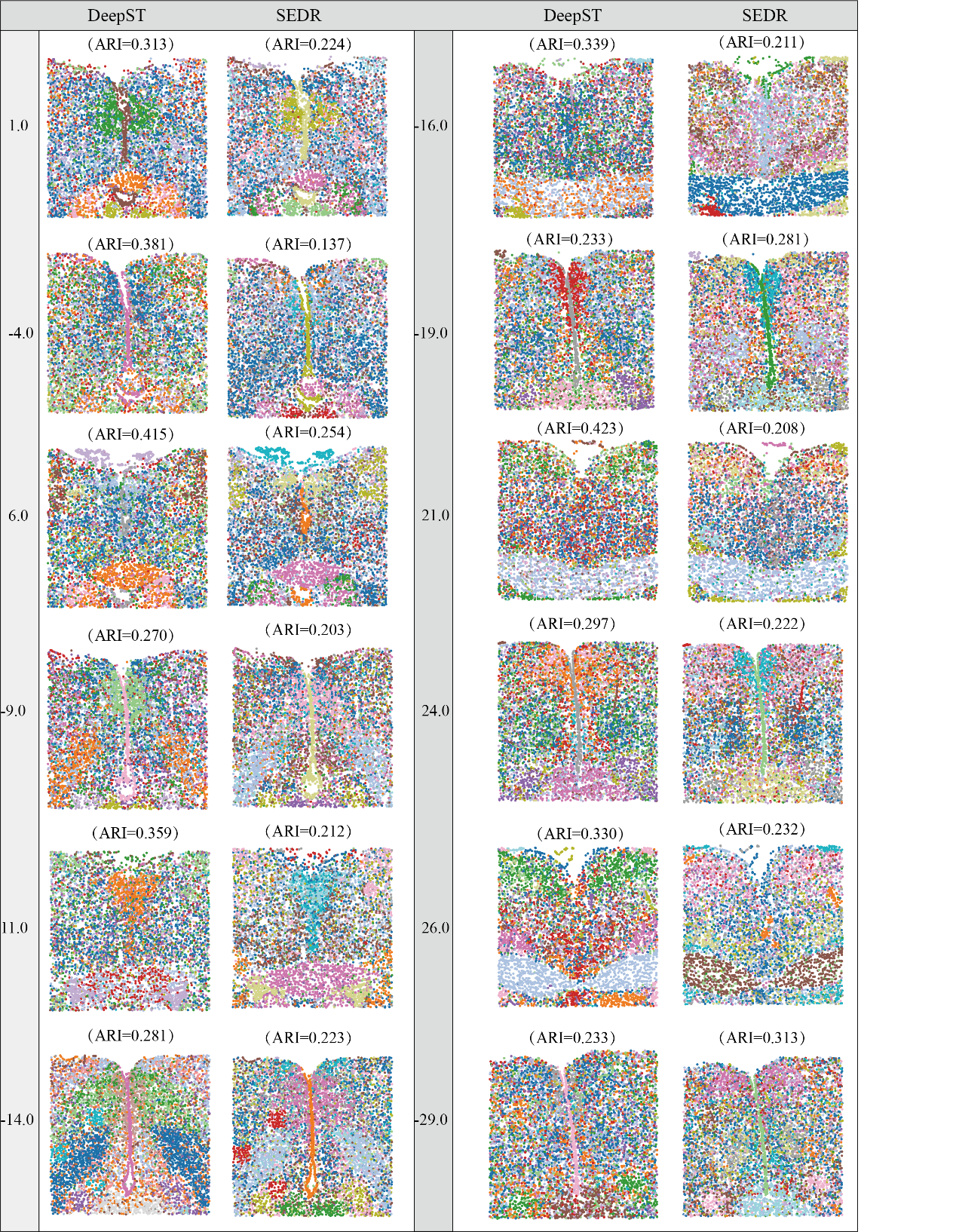


**Supplementary Figure 20.** DeepST and SEDR work on MERFISH datasets. DeepST and SEDR perform spatial domain identification on 12 MERFISH datasets and calculate ARI values, respectively.


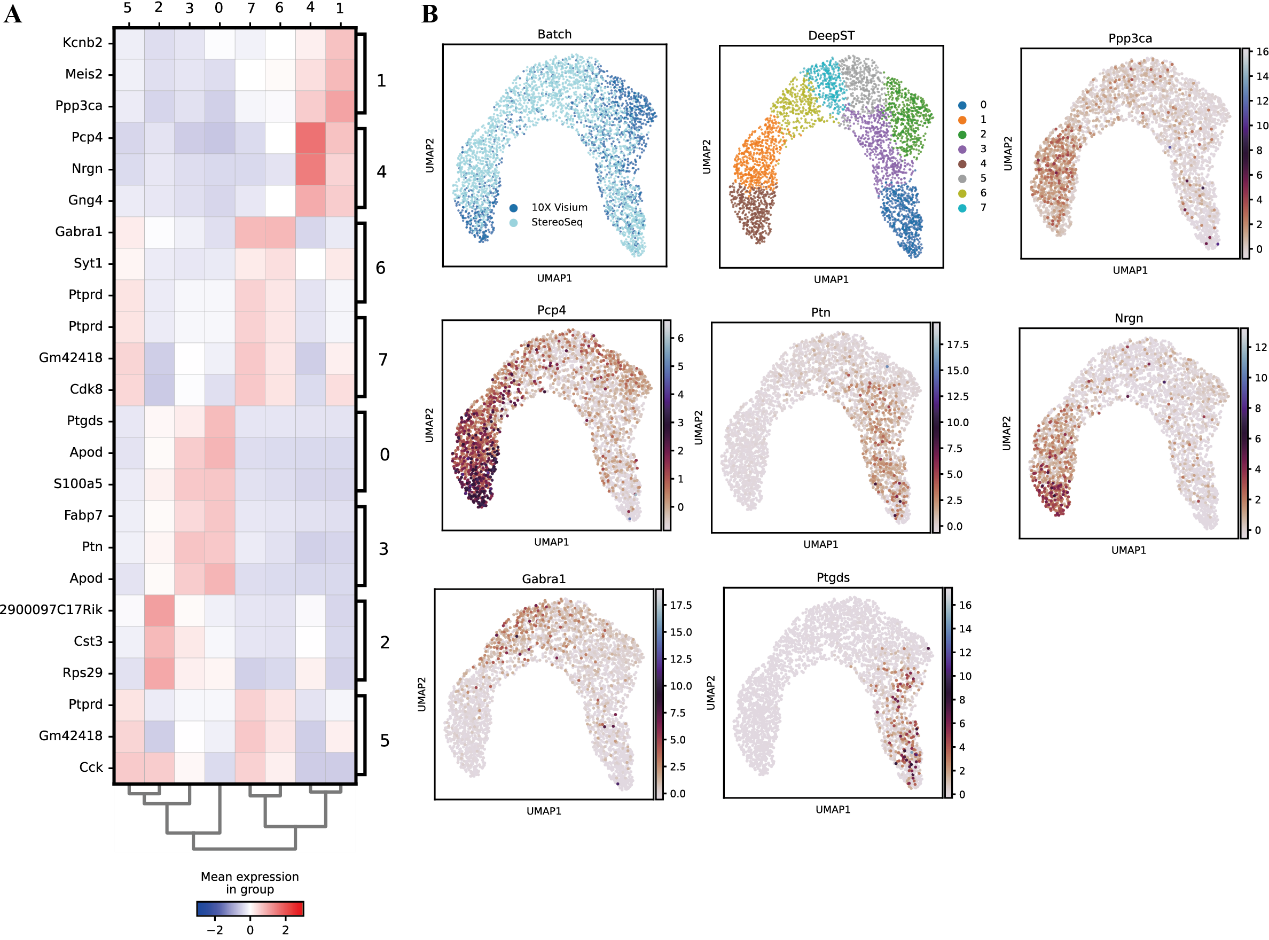


**Supplementary Figure 21.** Differential expression analysis on DeepST spatial domain. (**A**) Dotplot of the top 3 DEGs of all spatial domains on integrated mouse olfactory bulb (using 10× Genomics Visium and Stereoseq). (**B**) Scatter plot of spatial clustering generated by DeepST (including genes *Ppp3ca*, *Nrgn*, *Pcp4*, *Gabra1*, *Slc17a7*, *Ptgds* and *Ptn*).
